# Author Correction: QuasAr Odyssey: the origin of fluorescence and its voltage sensitivity in microbial rhodopsins

**DOI:** 10.1038/s41467-022-34147-2

**Published:** 2022-10-21

**Authors:** Arita Silapetere, Songhwan Hwang, Yusaku Hontani, Rodrigo G. Fernandez Lahore, Jens Balke, Francisco Velazquez Escobar, Martijn Tros, Patrick E. Konold, Rainer Matis, Roberta Croce, Peter J. Walla, Peter Hildebrandt, Ulrike Alexiev, John T. M. Kennis, Han Sun, Tillmann Utesch, Peter Hegemann

**Affiliations:** 1 Institute of Biology, Experimental Biophysics, Humboldt-Universität zu Berlin, Berlin, Germany; 2 Leibniz-Institut für Molekulare Pharmakologie, Berlin, Germany; 3 Department of Physics and Astronomy, Biophysics of Photosynthesis, Vrije Universiteit Amsterdam, Amsterdam, Netherlands; 4 Department of Physics, Molecular Biophysics and Nanomedicine, Freie Universität Berlin, Berlin, Germany; 5 Department of Chemistry, Physical chemistry/Biophysical Chemistry, Technische Universität Berlin, Berlin, Germany; 6 Institute of Physical and Theoretical Chemistry, Technische Universität Braunschweig, Braunschweig, Germany; 7 Department of Chemistry, Technische Universität Berlin, Berlin, Germany; 8 Present Address: Faculties of Medicine and Science, Brain Research Institute, University of Zurich, Zurich, Switzerland

**Keywords:** Molecular biophysics, Biological fluorescence, Biophysical chemistry, Membrane potential

Correction to: *Nature Communications* 10.1038/s41467-022-33084-4, published online 20 September 2022

The original version of this Article contained an error in the Abstract, which incorrectly read “These proteins named QuasArs have been used for imaging membrane voltage changes in cell cultures and small animals, but they could not be applied in living rodents.”. The correct version replaces this sentence with “These proteins named QuasArs have been used for imaging membrane voltage changes in cell cultures and small animals. However due to the low fluorescence intensity, these constructs require use of much higher light intensity than other optogenetic tools.”. This has been corrected in both the PDF and HTML versions of the Article.

