# Supplementary material for: QuasAr Odyssey: the origin of fluorescence and its voltage sensitivity in microbial rhodopsins
Source: Nat Commun. 2022 Sep 20;13:5501. doi: 10.1038/s41467-022-33084-4 (PMC9489792; doi:10.1038/s41467-022-33084-4)
Supplement: Supplementary file 1 — Supplementary Information [file 41467_2022_33084_MOESM1_ESM.pdf]

Supplementary Information for

QuasAr Odyssey:
The Origin of Fluorescence and its Voltage Sensitivity in Microbial Rhodopsins

Arita Silapetere, Songhwan Hwang, Yusaku Hontani, Rodrigo G. Fernandez Lahore, Jens Balke, Francisco Velazquez Escobar, Martijn Tros, Patrick E. Konold, Rainer Matis, Roberta Croce, Peter J. Walla, Peter Hildebrandt, Ulrike Alexiev, John T.M. Kennis, Han Sun, Tillmann Utesch, Peter Hegemann

Supplementary Table 1 Fluorescent Arch3 variants

Construct name	Mutations	Fluorescence, QY	Voltage sensitivity
Arch3 D95N[1]	Arch3 D95N	$4 \cdot 10^{-4}$	~60% per 100 mV
Arch3 D95N-D106E[2]	Arch3 D95N-D106E		~30% per 100 mV
Archer1[3]	Arch3 D95E-T99C	$3 \cdot 10^{-3}$	~ 85% per 100 mV
Arch5[4]	Arch3 V59A-P60L-D95E-T99C-P196S	$8.7 \cdot 10^{-3}$	*E.coli* assay, pH sensitive
Arch7[4]	Arch3 V59A-P60L-D95E-T99C-P196S-D222S-A225C	$1.2 \cdot 10^{-2}$	*E.coli* assay, not pH sensitive
QuasAr1[5]	Arch3 P60S-T80S-D95H-D106H-F161V	$8 \cdot 10^{-3}$	~ 30% per 100 mV
QuasAr2[5]	Arch3 P60S-T80S-D95Q-D106H-F161V	$4 \cdot 10^{-3}$	~ 90% per 100 mV
QuasAr3[6]	QuasAr2 K171R		~ 50% per 100 mV
paQuasAr3[6]	QuasAr3 V59A	1,1 x QuasAr3	~ 25% per 100 mV
Archon1[7]	Arch3 T20S-G41A-V44E-P60S-T80P-D86N-D95Q-D106H-A136T-F161V-T183I-L197I-G241Q	Brightness ~2-fold higher than QuasAr2	~ 80% per 100 mV
Archon2[7]	Arch3 T56P-P60S-T80P-D95H-T99S-T116I-F161V-T183I-L197I-A225C	Brightness ~3.5-fold higher than QuasAr1	~ 20% per 100 mV

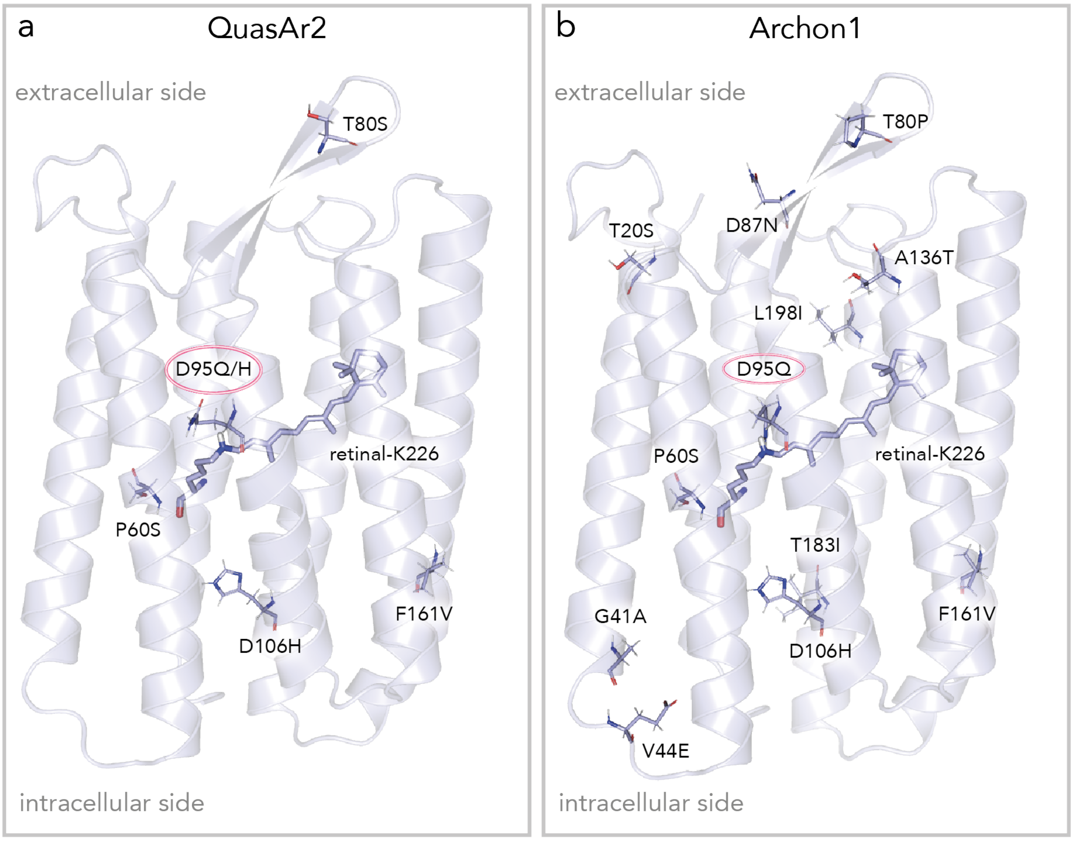

Supplementary Fig. 1 Homology model of QuasArs[5] and Archon1[7] based on crystal structure of archaerhodopsin-2 (PDB entry 3WQJ; 1.8 Å-resolution)[8]

(a). QuasAr1 carries a D95H mutation, where a negatively charged residue is replaced by a positive one. QuasAr2 carries a D95Q mutation, where a negatively charged residue is replaced by an neutrally charged residue. The D95 is the primary proton acceptor in Arch3 (D85 in BR). For both QuasArs, the proton donor D106H also carries a mutation where negatively charged residue is replaced by positively charged (D96 BR). QuasAr1 shows higher fluorescence quantum yield of $0.8 \cdot 10^{-3}$ with voltage sensitivity ΔF/F= 33% per 100 mV. QuasAr2 shows a lower fluorescence quantum yield of 0.4 $\cdot 10^{-3}$, but a higher voltage sensitivityΔF/F= 90% per 100 mV (640 nm excitation)[5].

(b) Archon1 is based on QuasAr2 and carries the counterion D95Q mutation where a negative charge is replaced by neutrally charged residue. Also, the proton donor D106H mutation is conserved. Brightens of the construct in mammalian cell culture was improved 2.4-fold. The fluorescence increase reported ΔF/F = 81% per 100mV (640 nm excitation)[7].

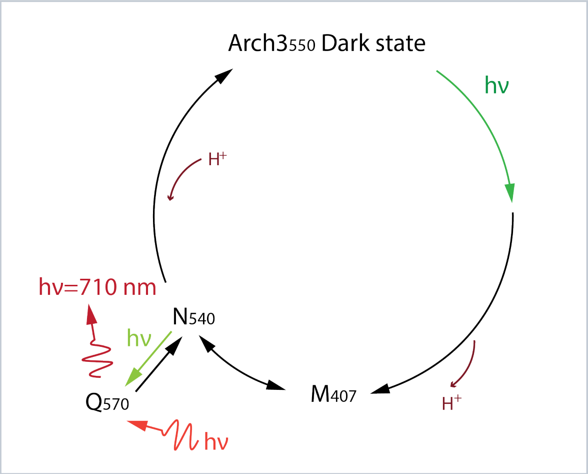

Supplementary Fig. 2 Arch3 Photocycle adapted from Maclaurin et al. 2013[9]

The Arch3 photocycle is excited with green (550 nm) light. It is proposed that voltage controls the equilibrium between the M and N states. Additional green light (540 nm) absorbed by N-state brings it to a side reaction where Q state is formed. Orange light (570nm) absorption by the Q state is re-emitted as fluorescence with a maximum of around 710 nm.

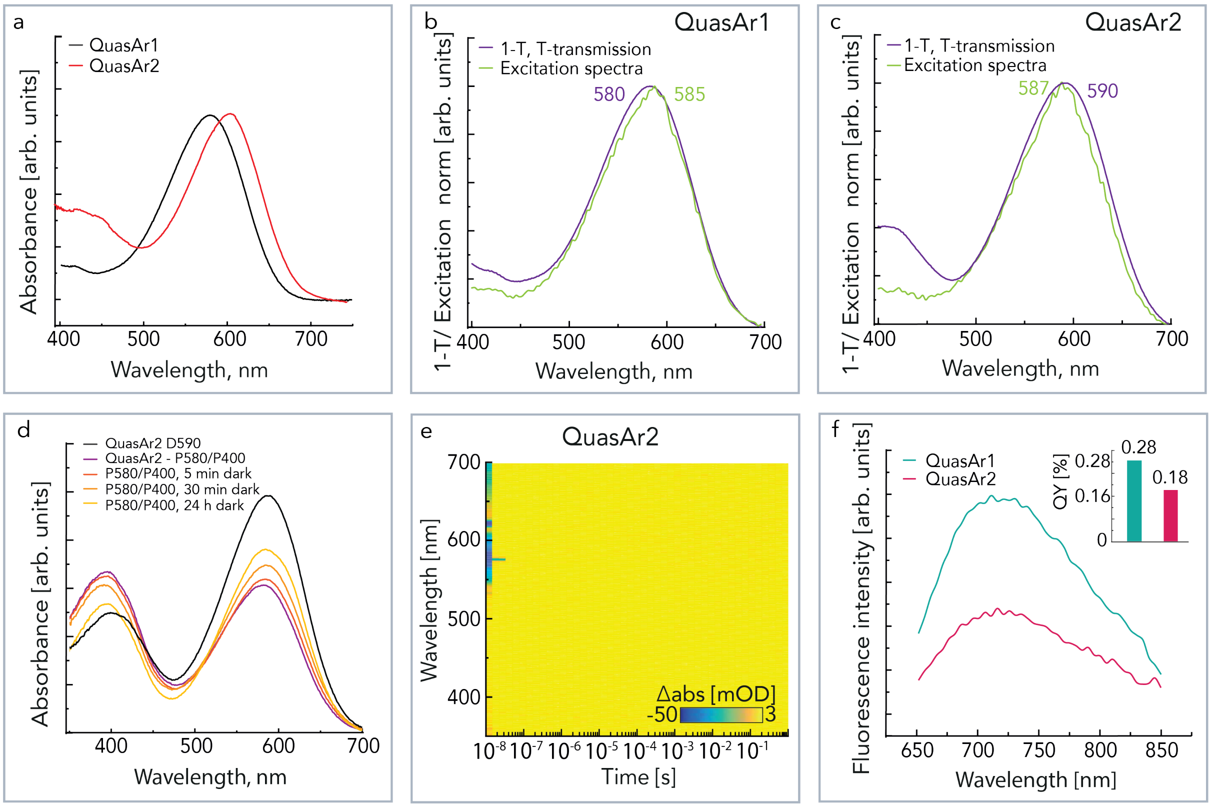

Supplementary Fig. 3 Characterization of QuasAr spectroscopical properties

(a) The absorption spectra of QuasAr1 (λ_{max}=580nm) and QuasAr2 (λ_{max}=590nm). (b,c) The (1-T) spectrum and the excitation spectrum (detected at 710 nm) are normalized at the peak. In QuasAr1 (b), the excitation spectrum is slightly red-shifted, but the difference is very minor. In QuasAr2 (c), the excitation spectrum displays a slight blue-shift. (d) Steady-state absorption of thermal recovery of QuasAr2 dark state. The dark state (D590; black curve) absorption maximum is at 590nm. After illuminating the sample with 625 nm, the blue-shifted (P580; purple curve) and UV absorbing species appear (P400; purple curve). To trace the recovery of the dark state absorption in the dark, QuasAr2 absorption spectra were recorded after 5 min (red curve), 30 min (orange curve), and 24h (yellow curve). (e) With flash photolysis spectroscopy, we recorded transient absorption spectra (TAS) in the spectral region of 350 nm -720 nm and in the time range between 10 ns up to 10 s. The excitation laser was tuned to 580 nm. Changes in RSB proximity would result in transient changes in the absorption spectra. However, we did not notice any evident formation of photo intermediates in the reconstructed 3D plot of the TAS. This implies that the transitions between the photoproducts are highly inefficient and consequential spectral changes are not detectable in single flash experiments. (f) The fluorescence QY of QuasAr1 (610 nm excitation) and QuasAr2 (620 nm excitation) measured in this study are lower than reported in Hochbaum et al. 2014[5]. Source data are provided in the Source Data file (Figure S3, Figure S3e).

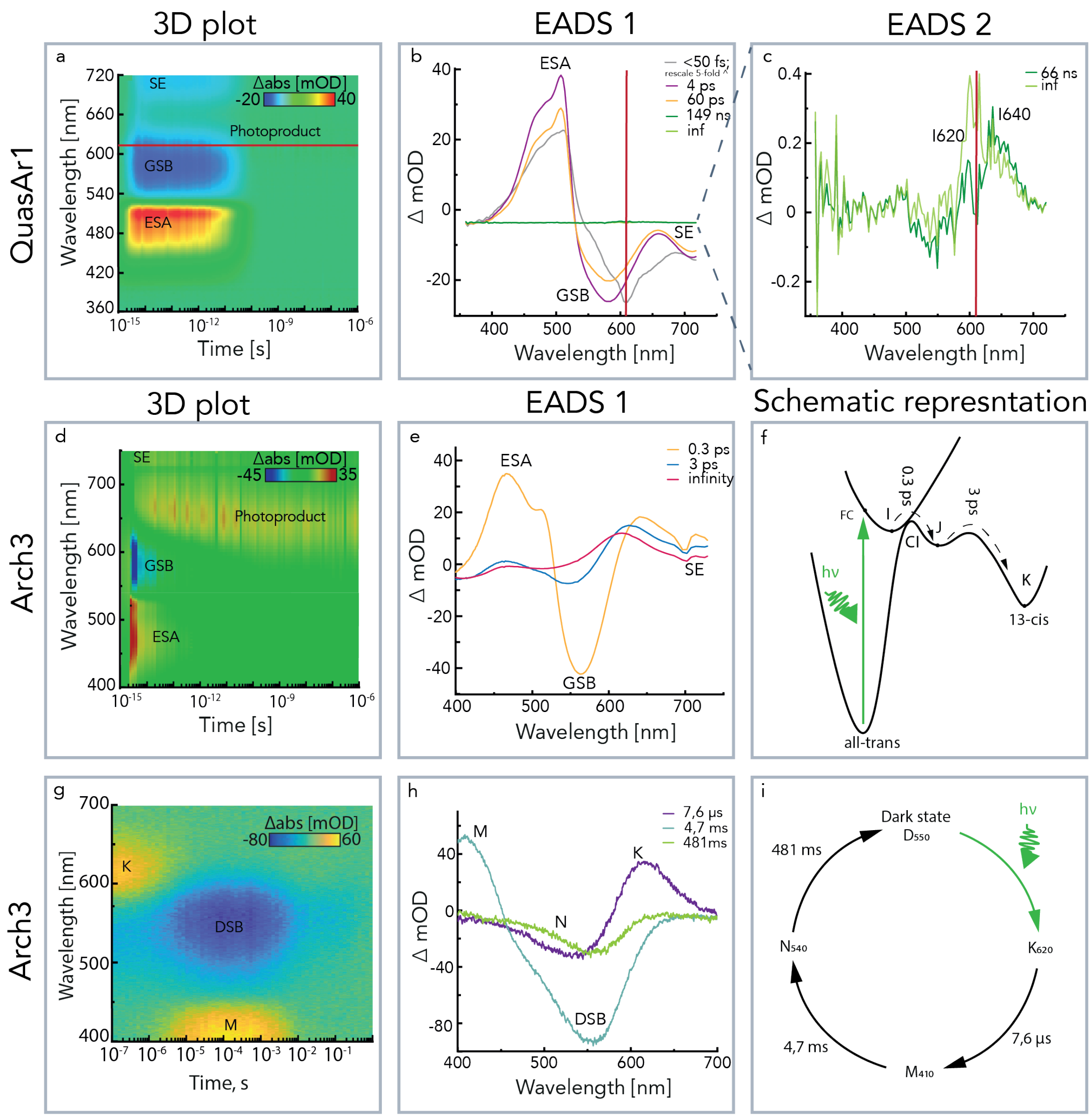

Supplementary Fig. 4 Transient absorption spectra of QuasAr1 and Arch3

(a-c) The transient absorption spectra in the time range of fs - µs were measured of QuasAr1 using pump-probe spectroscopy. The sample was excited with a laser tuned to 610 nm. Here, the excited state dynamics of QuasAr1 in the initial dark state D580 are shown. (a) The reconstructed 3D plot of transient absorption spectra (b) Evolution associated difference spectra (EADS) and (c) EADS of close up in the formed photointermediates. The rapid decay from the Franck Condon region was determined $\tau_0 \approx 3$ fs. As this value is below the IRF, it cannot be resolved properly. Also, in this time window, we see artifacts due to the mixing of the pump/probe beams, which results in disturbed difference spectra.

(d-f) The transient absorption spectra of Arch3 in the time region from fs to µs were measured using pump-probe spectroscopy. The sample was excited with a laser tuned to 580 nm. Here, the excited-state dynamics of Arch3 are shown as (d) reconstructed 3D plot of TAS and (e) EADS. In the upper right corner (e) the time constants of photointermediates are indicated. The scheme of excited state dynamics is shown in panel (f).

(g-i) The spectral evolution in the time range between 100 ns and 1 s was further studied with flash photolysis after sample excitation at 530 nm. The resulting reconstructed 3D plot (g) and EADS (h) are shown in the lower panels. The observed spectral kinetics provide information about the Arch3 photocycle (i). The global fit resulted in three time components that are assigned to three photointermediates – K, M, and N. In the red-shifted K state (620 nm) the protein comprises 13-*cis* retinal. The K state decays in 7,6 µs and the M state is formed. With the population of the M state, the proton from the RSB is transferred to proton acceptor D95 and is indicated by the blue-shifted absorption spectra (410 nm). Upon 4,7 ms the RSB is reprotonated from proton donor D106 and results in N state (540 nm). The N state is long-lived, and the dark state is recovered in 481 ms. Upon recovery of the dark state, the D106 is reprotonated from the cytoplasmic side and retinal backisomerizes to all-*trans*. Source data are provided in the Source Data file (Figure S4a - h).

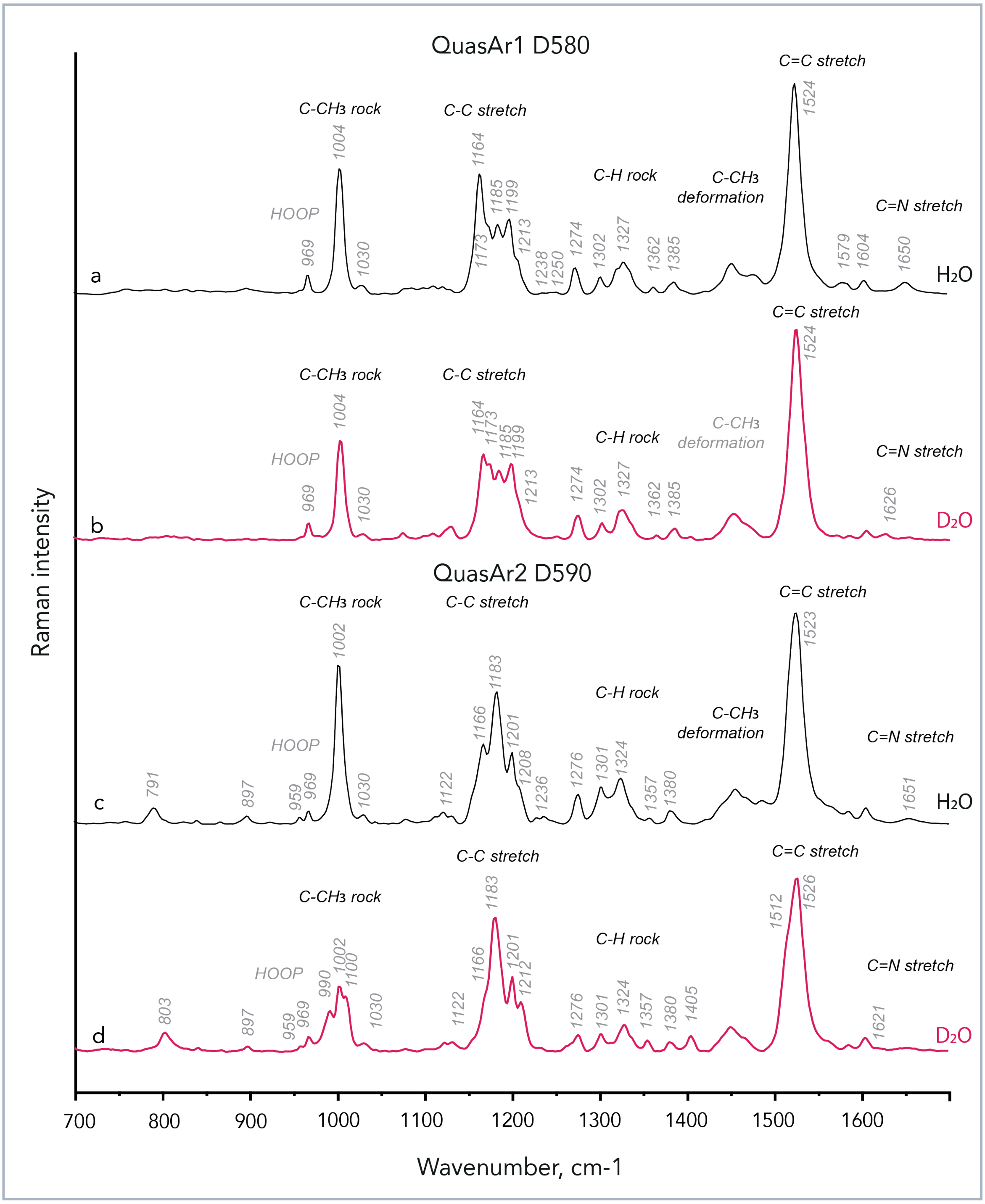

Supplementary Fig. 5 Pre-resonance Raman spectra of QuasAr1 and QuasAr2

Raman spectra were recorded with excitation under pre-resonance conditions (1064 nm, 640mW). The sample was purified in SMA nanodiscs. The QuasAr1 and QuasAr2 spectra are shown in (a) and (c), respectively. Additionally, spectra of QuasAr1 (b) and QuasAr2 (d) in D_2O are shown to support the assignment of modes associated with the Schiff base. From the C-C stretching region (fingerprint region), we can conclude that in QuasAr1 the prevailing chromophore configuration is all-*trans* (1164 and 1199 cm^{-1}), and in comparison, QuasAr2 shows an increase in signals attributable to the 13-

cis-15-*syn* retinal isomer (1166 and 1186 cm^{-1}). An additional indicator of a 13-*cis*-15-*syn* conformation is the HOOP mode at 791 cm^{-1}. The C=C stretching mode frequency displays an inverse relationship with the absorption maximum, but in this case, the signal appears to be more complex[10,11] The two constructs show a similar peak frequency at 1523/1524 cm^{-1}, but their absorption spectra in the UV-Vis region are shifted by 10 nm. Additionally, both constructs have different retinal isomer compositions with closely overlapping C=C stretch frequency, suggesting that the isomers present also display strongly overlapping absorption bands. From the C=N stretching, we observe that both of the construct chromophores are protonated, and they show strong hydrogen bonding of the Schiff base proton by the upshifted C=N frequency compared to Arch3[12]. Source data are provided in the Source Data file (Figure S5).

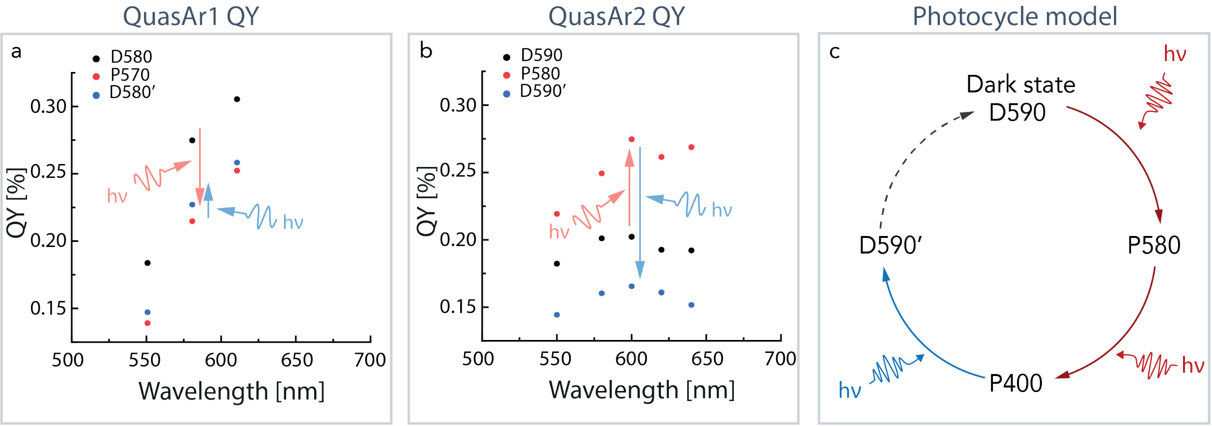

Supplementary Fig. 6 Excitation-dependent fluorescence QY

The excitation-dependent fluorescence QY is compared between the dark state (D580/D590, black dots) and subsequent photoproducts after red (P580, red dots) and blue (D590', blue dots) illumination. The determined fluorescence QY are shown for both constructs QuasAr1 (a) and QuasAr2 (b). The photocycle model (c) is taken from Fig. 1 for visual guidance. Source data are provided in the Source Data file (Figure S6).

Supplementary Table 2 Comparison of fluorescence QY in different photoproducts.

Construct	Retinal isomer composition	Fluorescence QY_{580}, %
QuasAr1-D580	**21% 13-*cis*, 79% all-*trans***	0.28
QuasAr1-P570	**18% 13-*cis*,** 14% 11-*cis*, 14% 9-*cis*, **54% all-*trans***	0.23
QuasAr1-D580'	**55% 13-*cis***, 14% 11-*cis*, 5% 9-*cis*, **26% all-*trans***	0.24
QuasAr2-D590	**47% 13-*cis*, 53% all-*trans***	0.20
QuasAr2-P580	**29% 13-*cis***, 16% 11-*cis*, 7% 9-*cis*, **48% all-*trans***	0.25
QuasAr2-D590'	**72% 13-*cis***, 3% 11-*cis*, 4% 9-*cis*, **21% all-*trans***	0.16

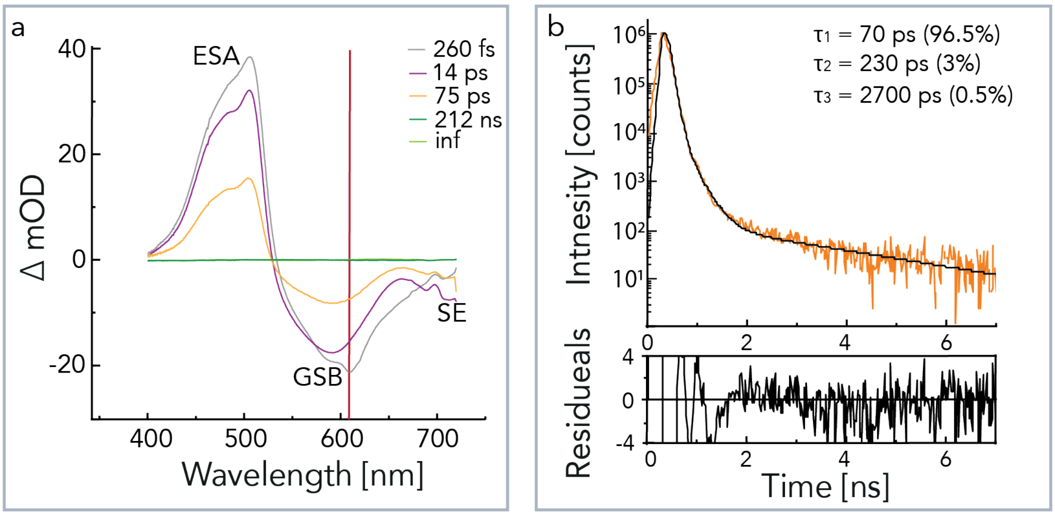

Supplementary Fig. 7 Archon1 excited-state dynamics and fluorescence lifetime

The panel (a) shows the excited state dynamics of solubilized Archon1 protein in 1.5% DDM and 0.3% CHS micelles, 150 mM NaCl 50mM Tris-HCl, pH 8.0 measured with fs pump-probe experiments with 620 nm excitation. The excited state decays with two time constants 14ps and 75ps. The ratio between τ_1 and τ_2 is 2:1. The panel (b) shows the fluorescence lifetime measured by FLIM at 19°C and excitation wavelength of 640nm. The first decay component of 14 ps is below the resolution of the FLIM setup, but the later time component ~75 ps is resolved. The fluorescence lifetime decay was fitted with three exponentials, where the major component (96.5% fractional amplitude) is 70 ps and matches the component observed by the excited state dynamic measurements with pump-probe. Source data are provided in the Source Data file (Figure S7).

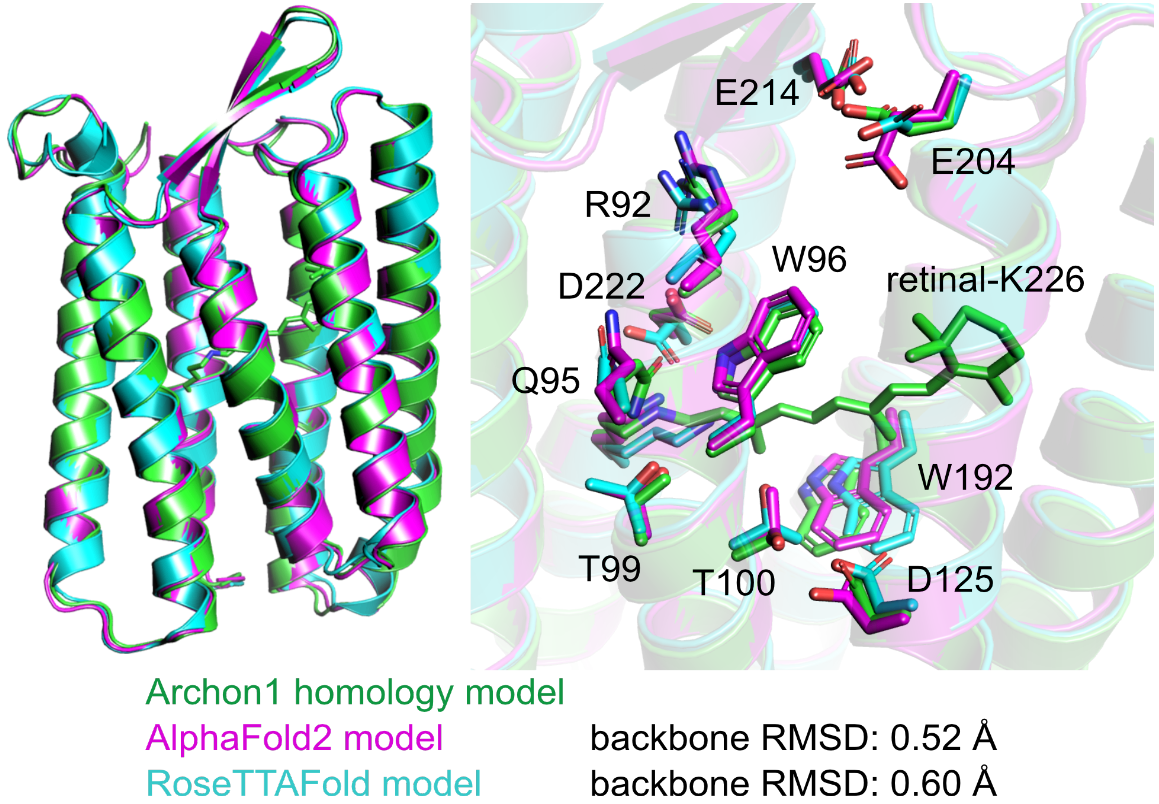

Supplementary Fig. 8 Comparison of Archon1 structures generated by homology modeling, AlphaFold2[13,14] and RoseTTAFold[15] predictions.

(Left) The predicted Archon1 models of CoLab Alphafold2 (magenta cartoon) and RoseTTAFold (cyan cartoon) are superimposed to the Archon1 homology model (green cartoon). (Right) Comparable orientation and position of the side-chains of the key residues are highlighted in the three models.

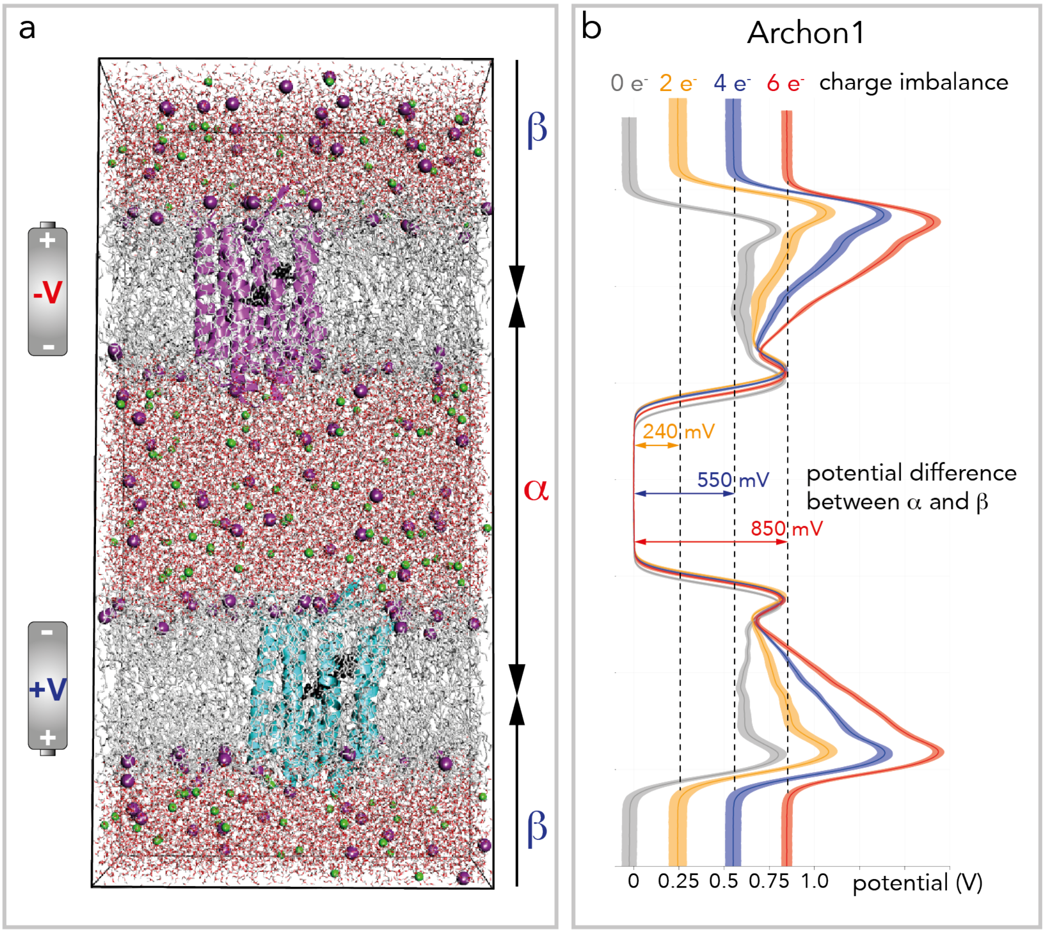

Supplementary Fig. 9 Computational electrophysiology

(a) Computational electrophysiology setup. Two lipid bilayers (grey sticks) generate two separated compartments (α and β) with a different number of explicit ions (K^+: purple spheres; Cl^-: green spheres). Proteins are colored according to the applied voltage (negative: magenta; positive: cyan). The retinal cofactor and water molecules are denoted as black spheres and red/white lines, respectively. (b) The membrane potential of Archon1 is generated by charge imbalance between the compartments α and β. The analysis was performed on six 200-ns simulations for each membrane voltage. The mean and standard deviation of the repetition of the simulations are represented as solid line and shade, respectively. The MD simulations were conducted at 303 K by fixing the protonation states of titratable sites at neutral pH. Source data are provided in the Source Data file (Figure S9b).

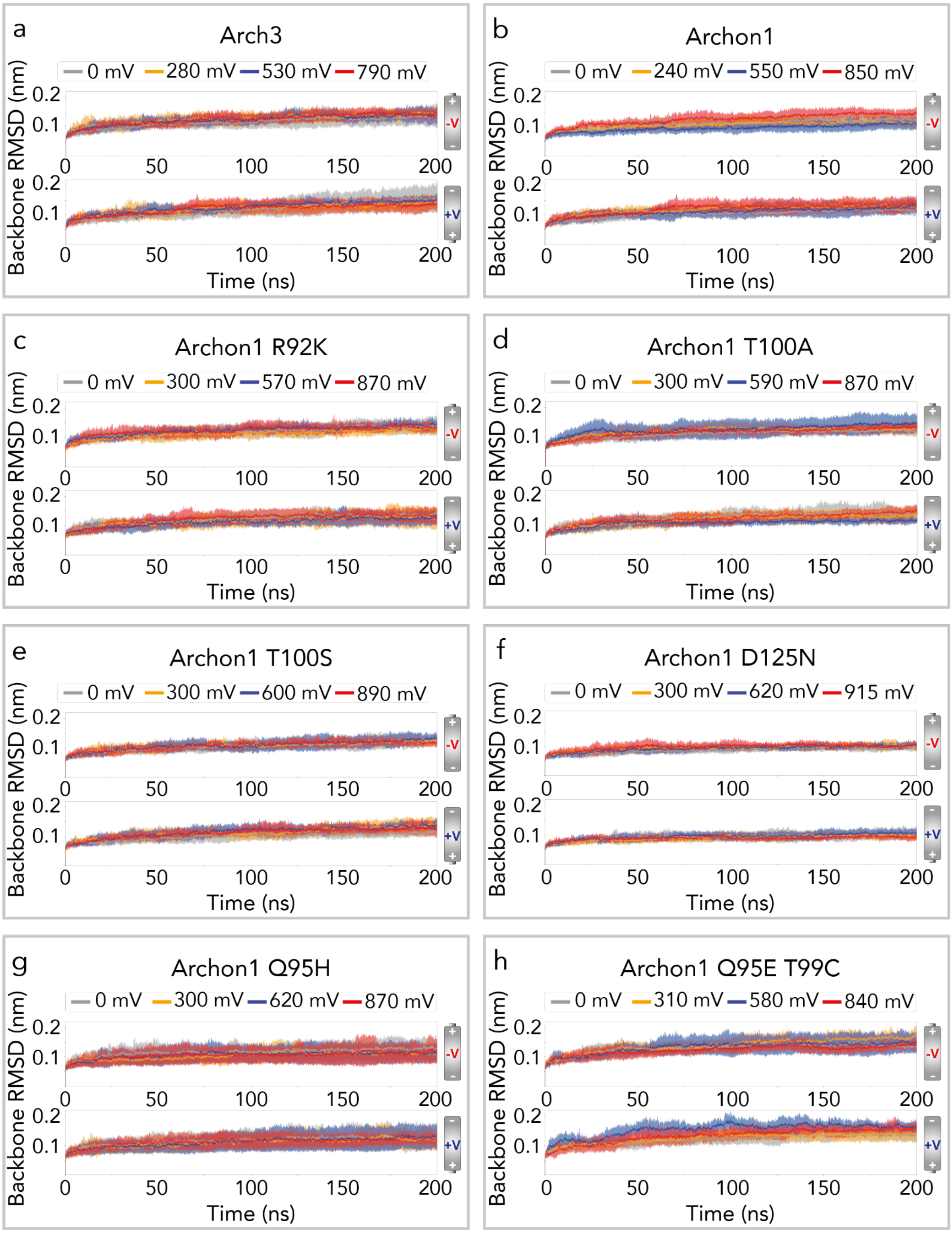

Supplementary Fig. 10 Backbone root mean square deviations (RMSDs)

RMSDs were calculated taking the equilibrated systems at 0 ns as reference. The solid line and shaded area represent the average and the standard deviation of RMSDs, respectively. For each simulation setup, six independent runs of 200 ns were performed. The MD simulations were conducted at 303 K by fixing the protonation states of titratable sites at neutral pH. Source data are provided in the Source Data file (Figure S10).

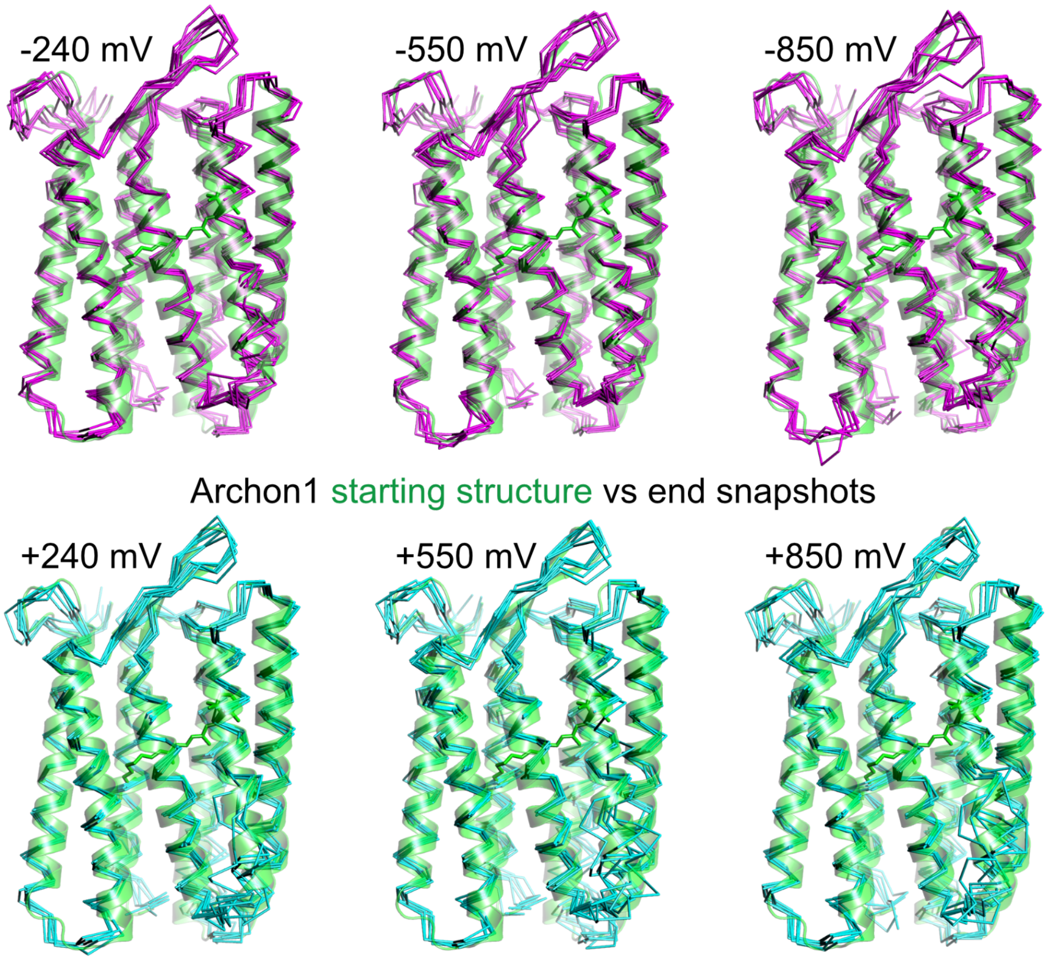

Supplementary Fig. 11 Comparison between the starting structure and end snapshots of the MD simulations of Archon1 performed at different transmembrane voltages

The end structures of Archon1 (ribbon representation) from the six simulations at different membrane voltages (magenta for negative, cyan for positive) are superimposed to the starting structure (green cartoon representation). The MD simulations were conducted at 303 K by fixing the protonation states of titratable sites at neutral pH.

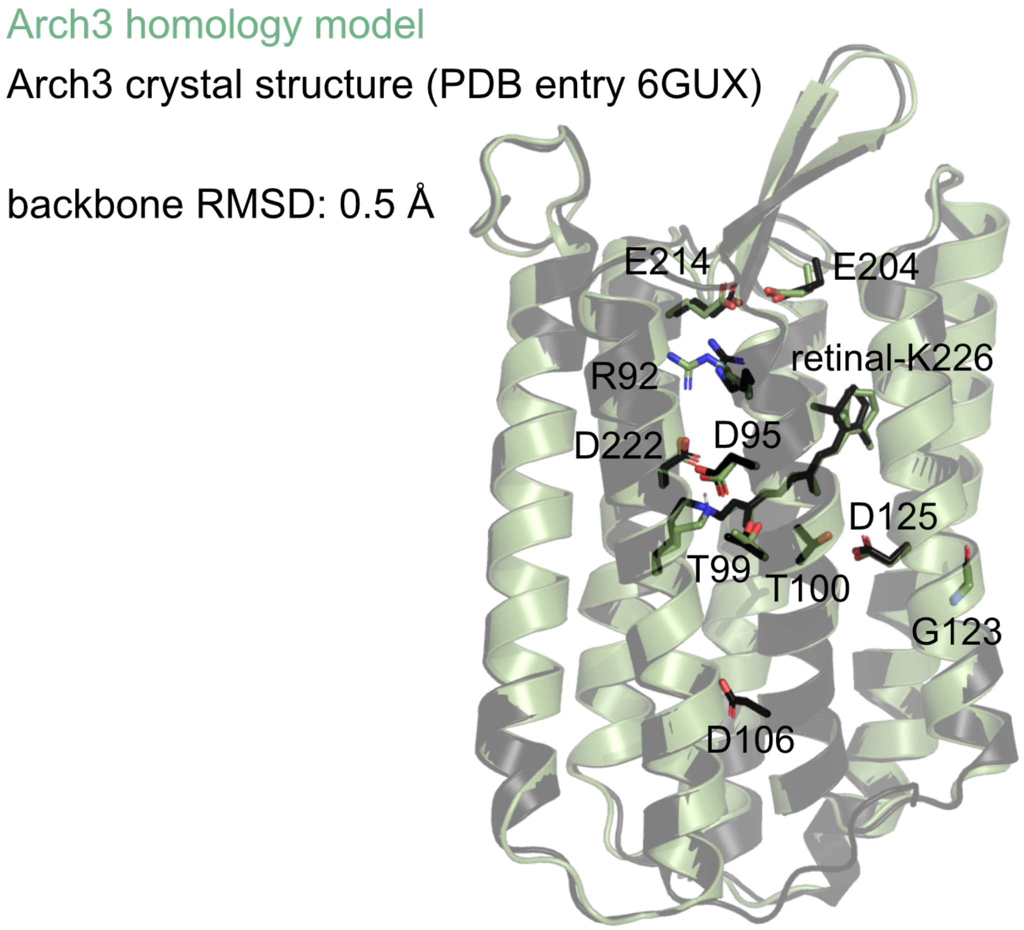

Supplementary Fig. 12 Comparison of the Arch3[16] crystal structure (black) and our homology model (green). The backbone RMSD between the Archon1 homology model based on the crystal structure of Arch2 (PDB entry 3WQJ) and the crystal structure of Arch3 (PDB entry 3GUX) is 0.5 Å.

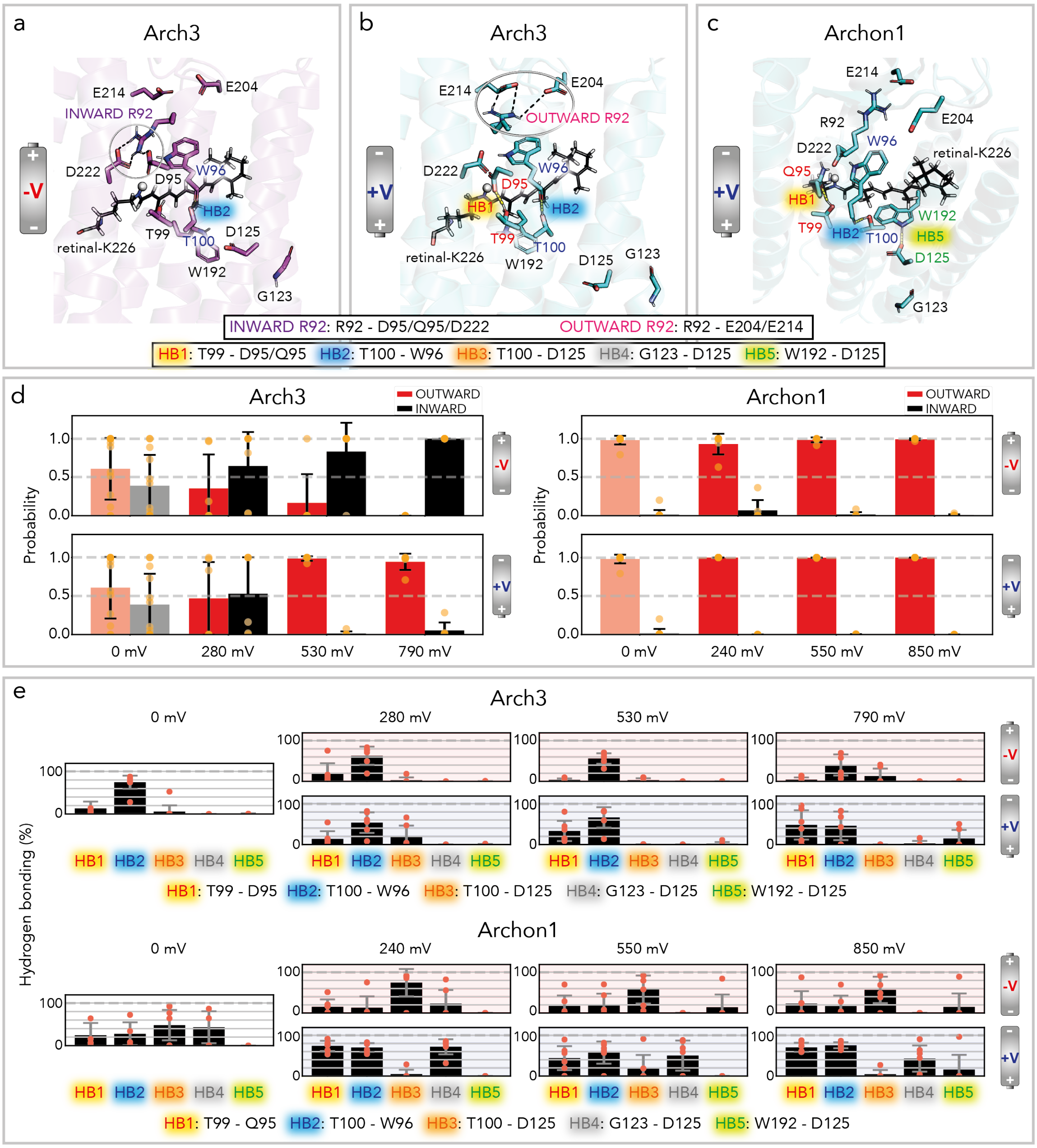

Supplementary Fig. 13 Voltage sensing in Arch3 and Archon1

(a, b) Voltage-induced conformations of Arch3. At a negative voltage, the key residue R92 interacts with D95 and D222 (inward R92) adjacent to the chromophore (a) while it flipped towards extracellular E204 and E214 at a positive voltage (outward R92) (b). This reorientation strongly affected the salt bridge and hydrogen bonding network around the retinal (HB1). (c, e) Hydrogen bonding interaction between indole N-H of W196 and D125 (HB5) in Archon1 was rarely found. (a-c) The proton at the retinal Schiff base is represented as a white sphere. (d) Voltage-dependent orientation of R92. (e) Voltage-dependent formation of hydrogen bonds (HB1: T99Hγ1 – D95Oδ1/2 / Q95Oε1, HB2: T100Hγ1 – W96O, HB3: T100Hγ1 – D125Oδ1/2, HB4: G125^{NH} – D125Oδ1/2, HB5: W192Hε1 – D125Oδ1/2). (d, e) The analysis was conducted on the last 100 ns of the trajectories. The

mean and standard deviation of the repetition of the simulations are represented as bar height and error bar, respectively. Each orange circle represents the data derived from the individual MD trajectories. The MD simulations were conducted at 303 K by fixing the protonation states of titratable sites at neutral pH. Source data are provided in the Source Data file (Figure S13d, e).

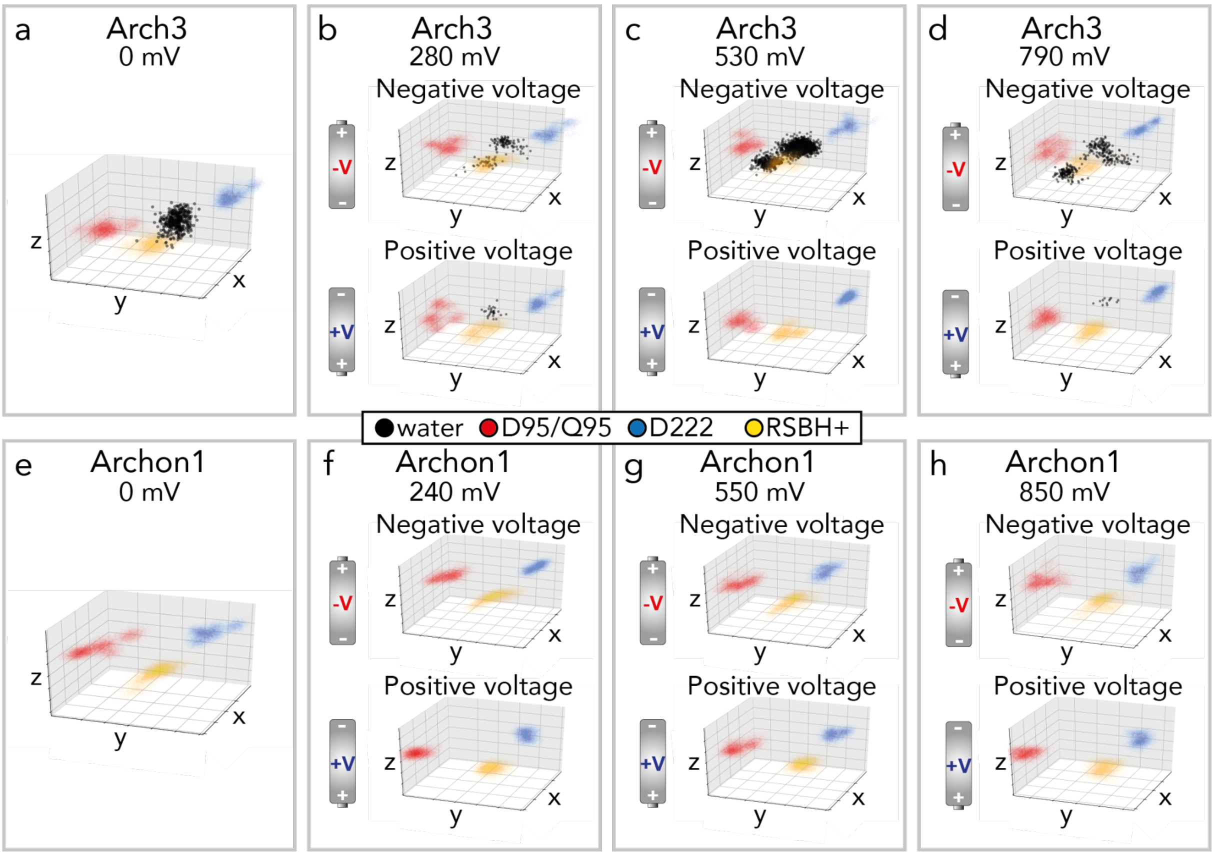

Supplementary Fig. 14 Water occupancy in Arch3 and Archon1

Water occupancy near the $RSBH^+$. The water oxygens within a sphere of 2 Å centered at the $RSBH^+$ are represented by black dots. The proton of the Schiff base, Cγ of D95 (Cδ of Q95 for Archon1), and Cγ of D222 are represented by yellow, red, and blue dots, respectively. All analysis was conducted after discarding the first half of each 200 ns trajectory to yield equilibrated dynamics. The MD simulations were conducted at 303 K by fixing the protonation states of titratable sites at neutral pH. Source data are provided in the Source Data file (Figure S14).

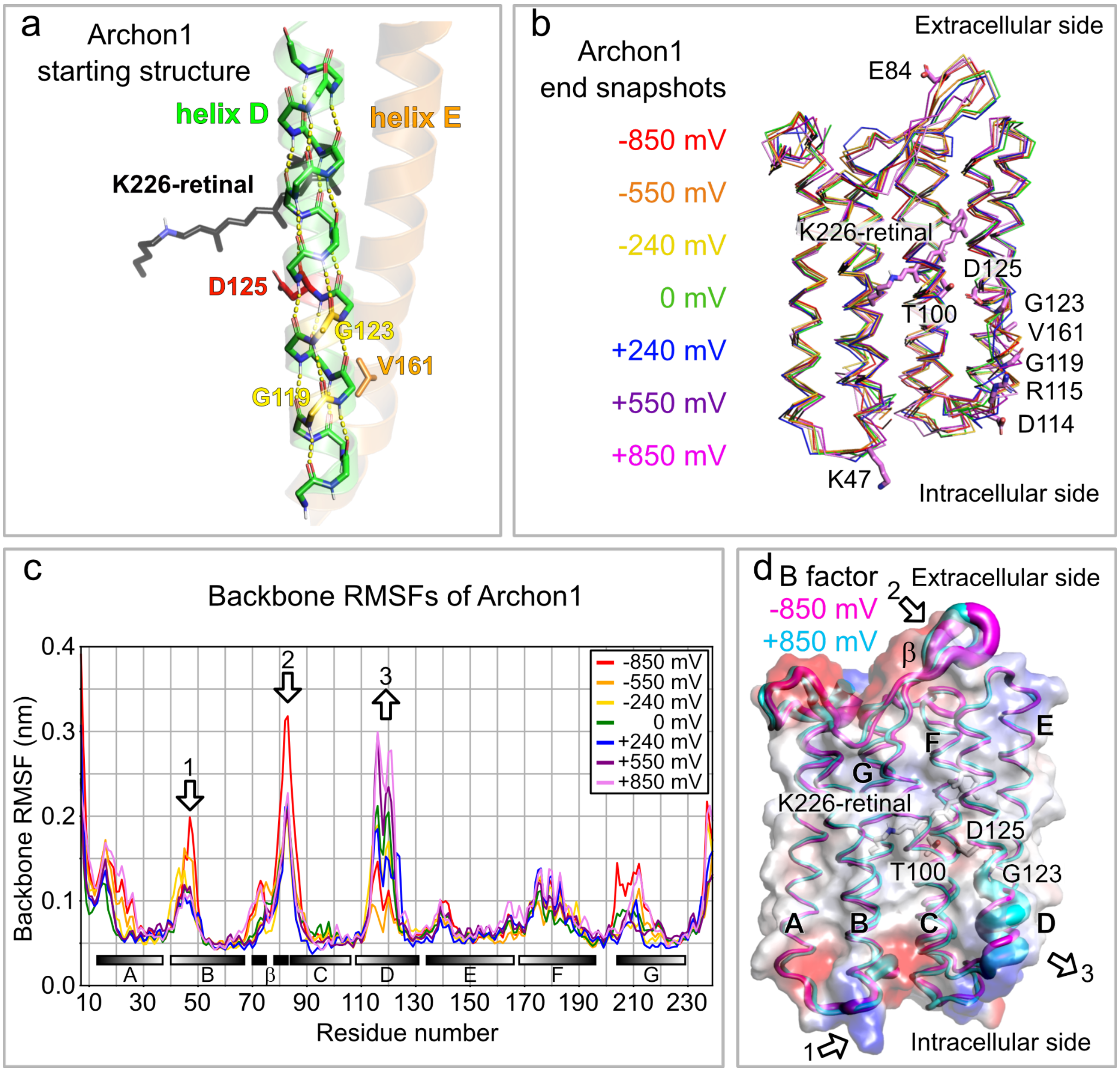

Supplementary Fig. 15 Voltage-dependent conformational changes of helix D in Archon1

(a) Hydrogen bonding network of helix D in Archon1 in the starting conformation of the MD simulation. (b) Superimposition of the end snapshots of Archon1 at different transmembrane voltages. (c) Backbone root mean square fluctuation (RMSF) of Archon1 derived from MD simulations at different transmembrane voltages. (d) Mapping of the B-factor derived from MD simulations on the structure of Archon1 at positive and negative transmembrane voltages. Thicker traces indicate higher B-factors. Positively and negatively charged residues are highlighted as blue and red surfaces on the electrostatic potential map, respectively. The MD simulations were conducted at 303 K by fixing the protonation states of titratable sites at neutral pH. Source data are provided in the Source Data file (Figure S15c).

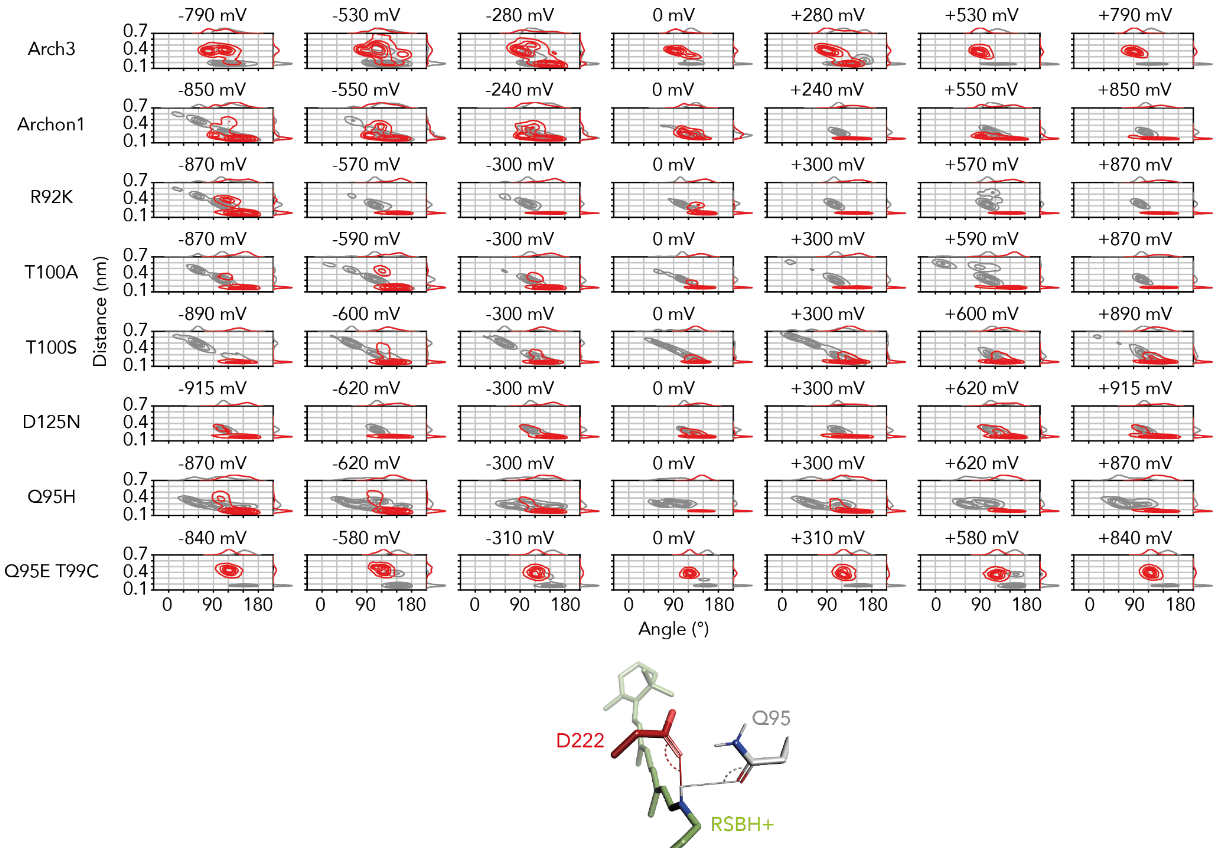

Supplementary Fig. 16 Interactions between RSBH$^+$ and the counter ions in Arch3, Archon1, and Archon1's variants

Distance and angle distributions between the RSBH$^+$ and D/Q/H/E95 at different membrane voltages are colored in grey. Distance and angle distributions between the RSBH$^+$ and D222 at different membrane voltages are colored in red. (lower panel) Minimum distances are calculated between the oxygen atoms (nitrogen atoms in the case of H95) of the side-chains (X95/D222) and the proton at the RSBH$^+$. Angles are defined between the RSBH$^+$, the corresponding oxygen atom (nitrogen in the case of H95) and the bound carbon atom (Cγ in D95; Cδ in Q95 and E95; Cε1 in H95). The analysis was conducted after discarding the first half of each 200-ns trajectory to yield equilibrated dynamics. The MD simulations were conducted at 303 K by fixing the protonation states of titratable sites at neutral pH. Source data are provided in the Source Data file (Figure S16).

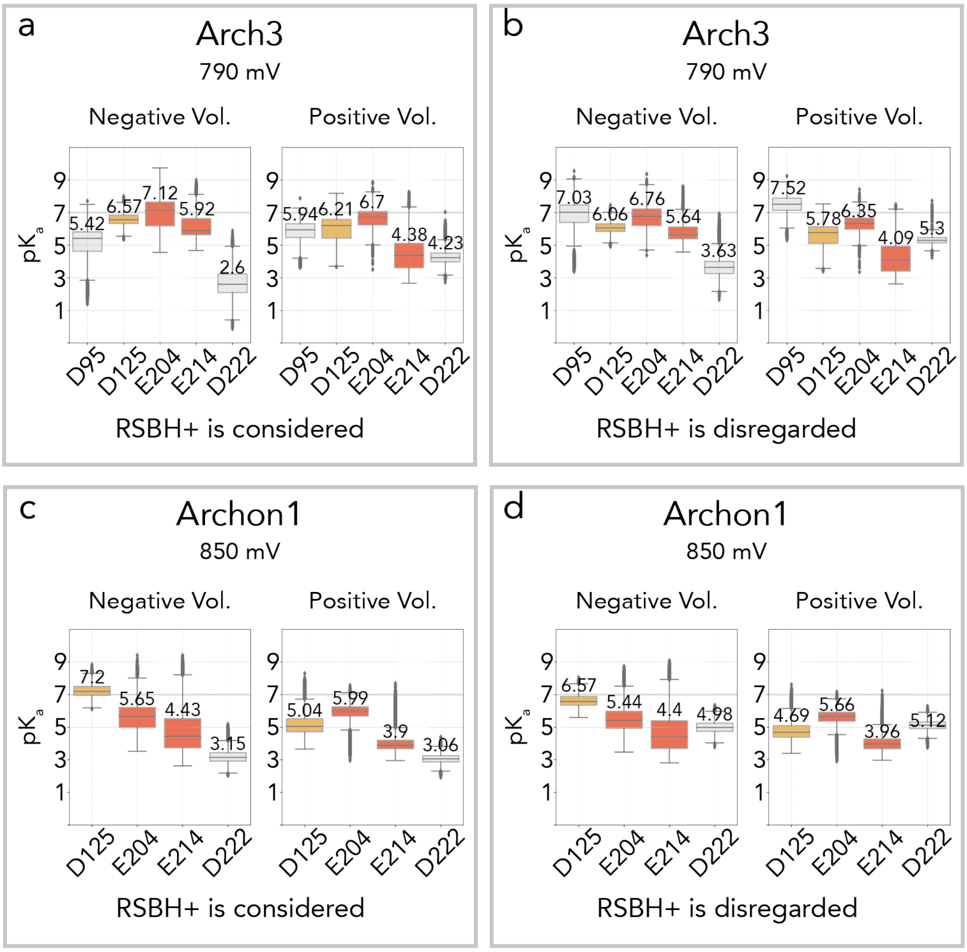

Supplementary Fig. 17 Calculated *pKa* values of key residues in Arch3 and Archon1

Calculated pK_a values of key titratable residues at different membrane voltages for Arch3 (a,b) and Archon1 (c,d). The pK_a values were calculated by considering the protonated retinal-K226 ($RSBH^+$) as a lysine (a,c) and by disregarding the protonated retinal (b,d). (a-d) Six 200-ns simulations were performed for each membrane voltage. All analysis was conducted after discarding the first half of each 200-ns trajectory to yield equilibrated dynamics. The upper and the lower bounds of box represent 75th and 25th percentiles, respectively. The median values are annotated in the box. The upper and the lower bounds of whiskers were defined by 75th percentile + 1.5 × (75th percentile – 25th percentile) and 25th percentile – 1.5 × (75th percentile – 25th percentile), respectively. The outliers are depicted as grey dots. (a) The reorientations of R92 modulate pK_a value of the ionizable residues near the proton of the retinal Schiff base (D95, E204, E214, D222). Outward reorientation of R92 at positive voltage increases the pK_a value of the counterions (from 5.42 to 5.94 for D95; from 2.6 to 4.23 for D222) and decreases the pK_a value of the proton release group (from 7.12 to 6.7 for E204; from 5.92 to 4.38 for E214). (b) Disregarding the retinal for the pK_a calculation showed an increase of the pK_a value of D95 to about 7. The MD simulations were conducted at 303 K by fixing the protonation states of titratable sites at neutral pH. Source data are provided in the Source Data file (Figure S17).

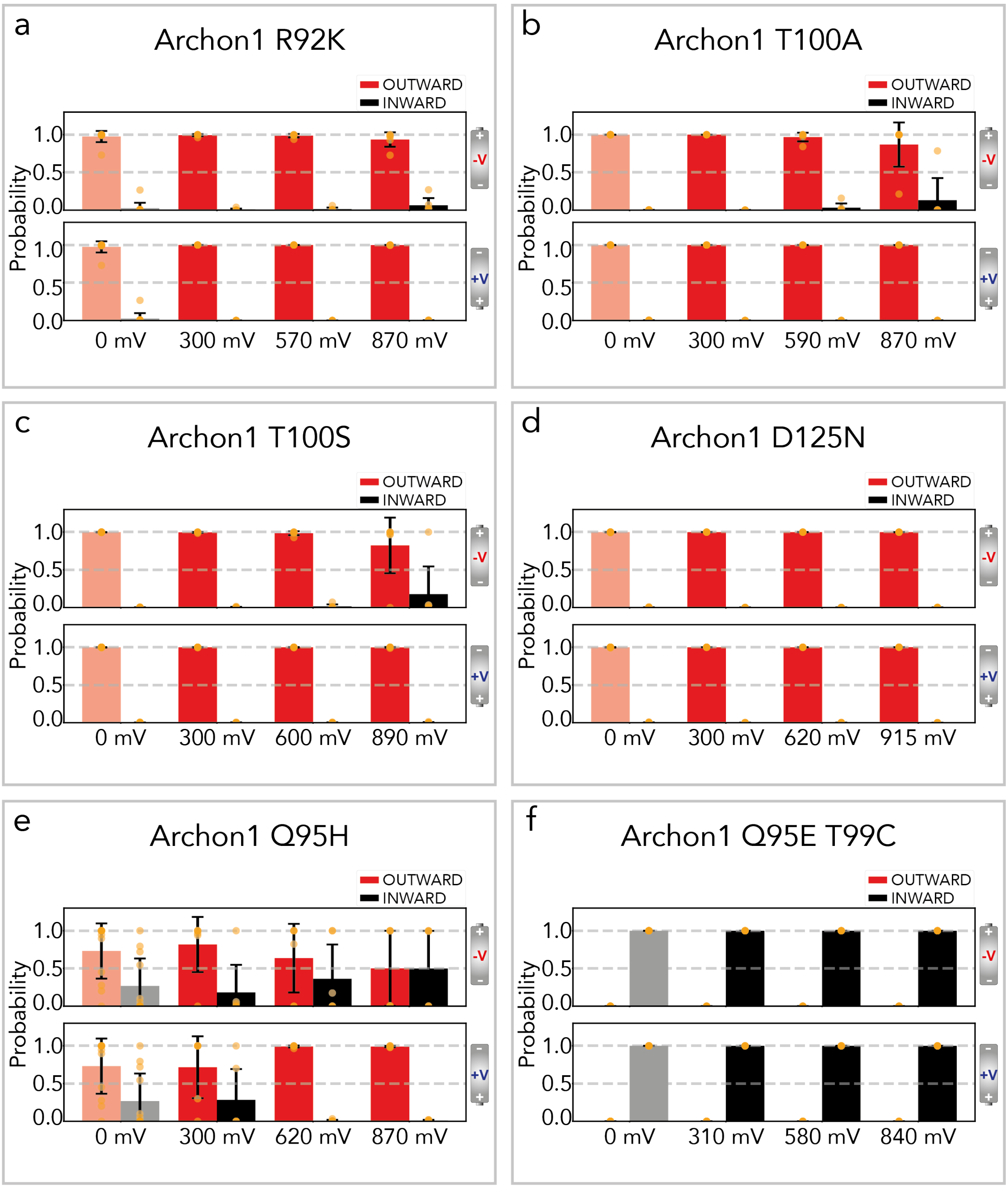

Supplementary Fig. 18 Probability of side-chain reorientation of R(K)92

Six 200-ns simulations were performed for each membrane voltage. All analysis was conducted after discarding the first half of each 200-ns trajectory to yield equilibrated dynamics. The side-chain reorientation of R/K92 to extracellular and intracellular directions are termed 'outward' and 'inward', respectively. The mean and standard deviation of the repetition of the simulations are represented as bar height and error bar, respectively. Each orange circle represents the data derived from the individual MD trajectories. The MD simulations were conducted at 303 K by fixing the protonation states of titratable sites at neutral pH. Source data are provided in the Source Data file (Figure S18).

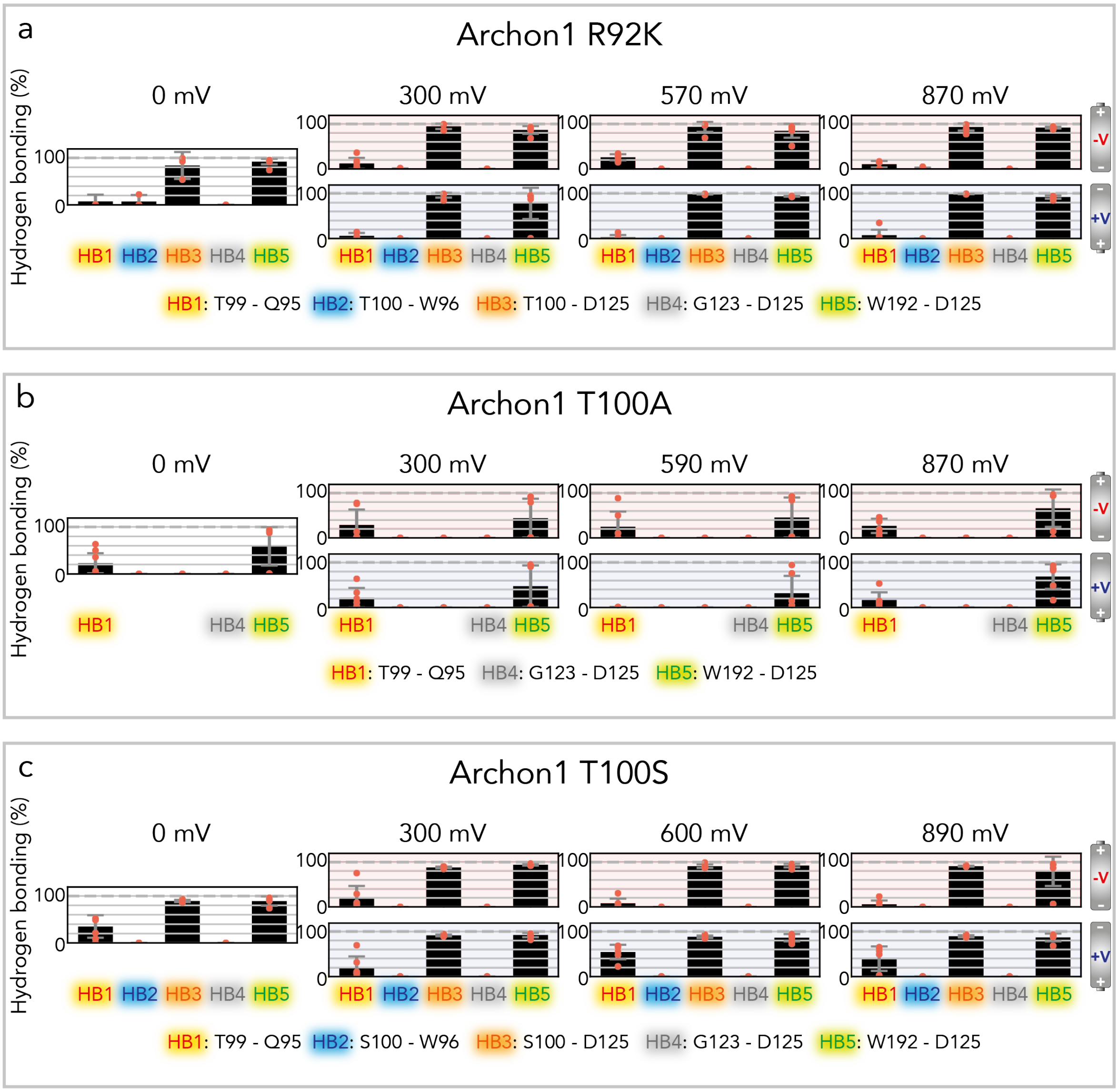

Supplementary Fig. 19 Hydrogen bonding network under different membrane voltages

HB1: T99Hγ1 - Q95Oε1, HB2: T100Hγ1/S100Hγ - W96O, HB3: T100Hγ1 - D125Oδ1/2, HB4: G125NH - D125 Oδ1/2, HB5: W192Hε1 - D125Oδ1/2. Six 200 ns simulations were performed for each membrane voltage. All analysis was conducted after discarding the first half of each 200 ns trajectory to yield equilibrated dynamics. The mean and standard deviation of the repetition of the simulations are represented as bar height and error bar, respectively. Each orange circle represents the data derived from the individual MD trajectories. The MD simulations were conducted at 303 K by fixing the protonation states of titratable sites at neutral pH. Source data are provided in the Source Data file (Figure S19).

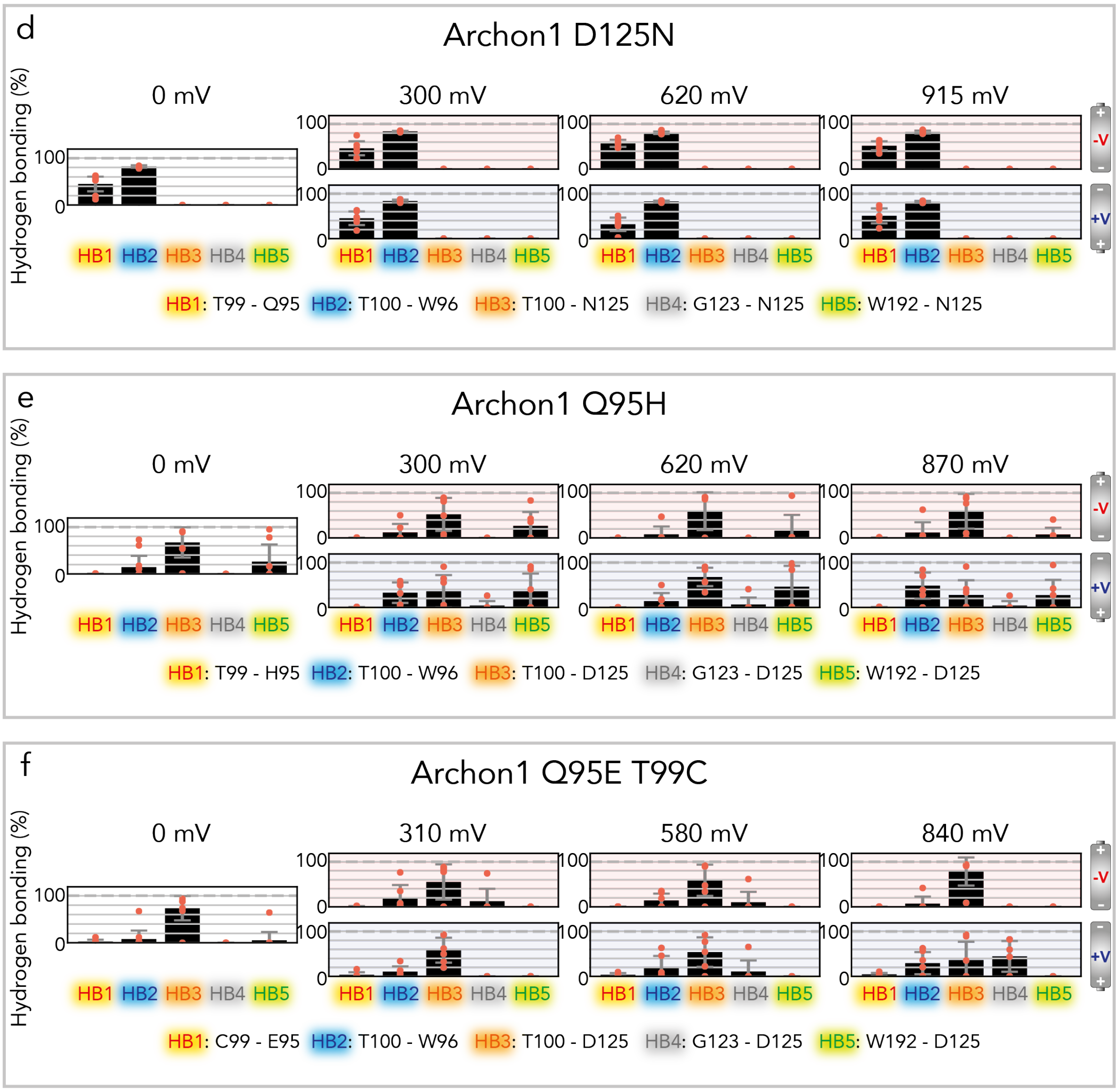

Supplementary Fig. 20 Hydrogen bonding network under different membrane voltages

HB1: T99Hγ1 / C99Hγ - Q95Oε1 / H95Nδ1/Nε2 / E95Oε1/2, HB2: T100Hγ1 - W96O, HB3: T100Hγ1 - D125Oδ1/2 / N125Oε1, HB4: G125NH - D125 Oδ1/2 / N125Oε1, HB5: W192Hε1 - D125Oδ1/2 / N125Oε1. Six 200 ns simulations were performed for each membrane voltage. All analysis was conducted after discarding the first half of each 200 ns trajectory to yield equilibrated dynamics. The mean and standard deviation of the repetition of the simulations are represented as bar height and error bar, respectively. Each orange circle represents the data derived from the individual MD trajectories. The MD simulations were conducted at 303 K by fixing the protonation states of titratable sites at neutral pH. Source data are provided in the Source Data file (Figure S20).

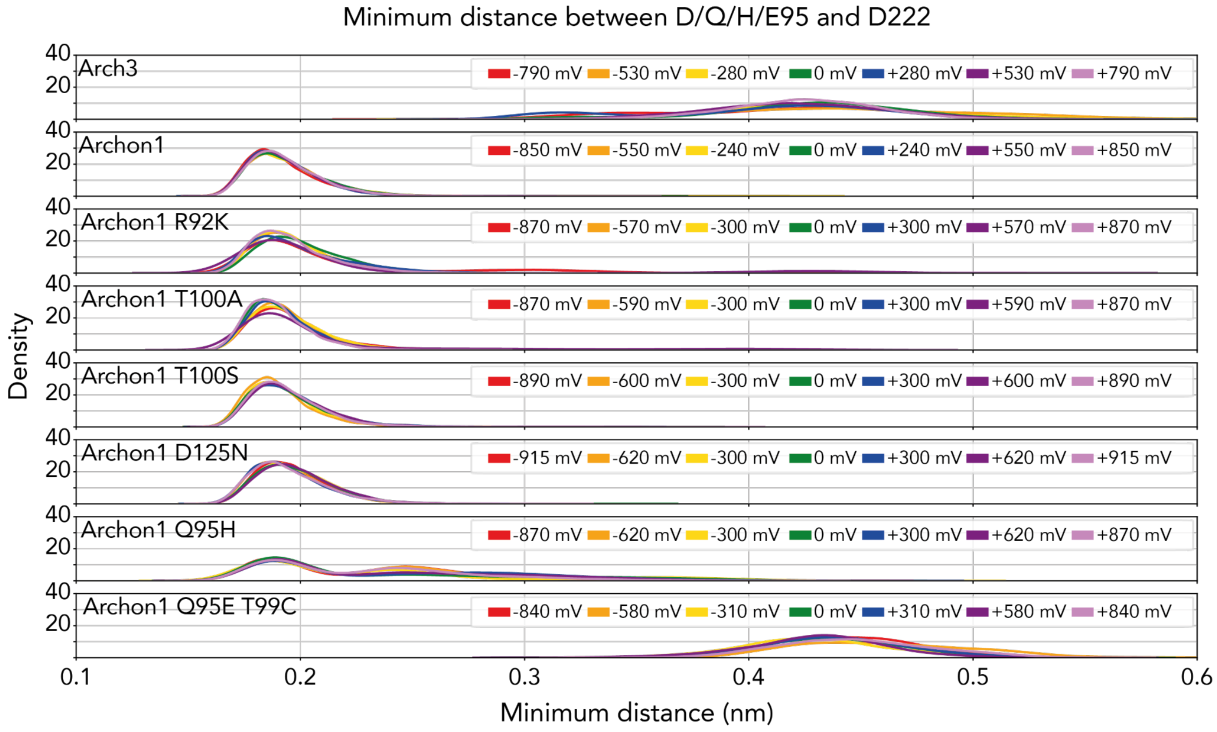

Supplementary Fig. 21 Minimum distance between D/Q/H/E95 and D222 in Arch3, Archon1, and Archon1's variants

Minimum distances between the side-chains of D/Q/H/E95 and the one of D222 at different membrane voltages. The analysis was performed on six 200-ns simulations for each membrane voltage. The MD simulations were conducted at 303 K by fixing the protonation states of titratable sites at neutral pH. Source data are provided in the Source Data file (Figure S21).

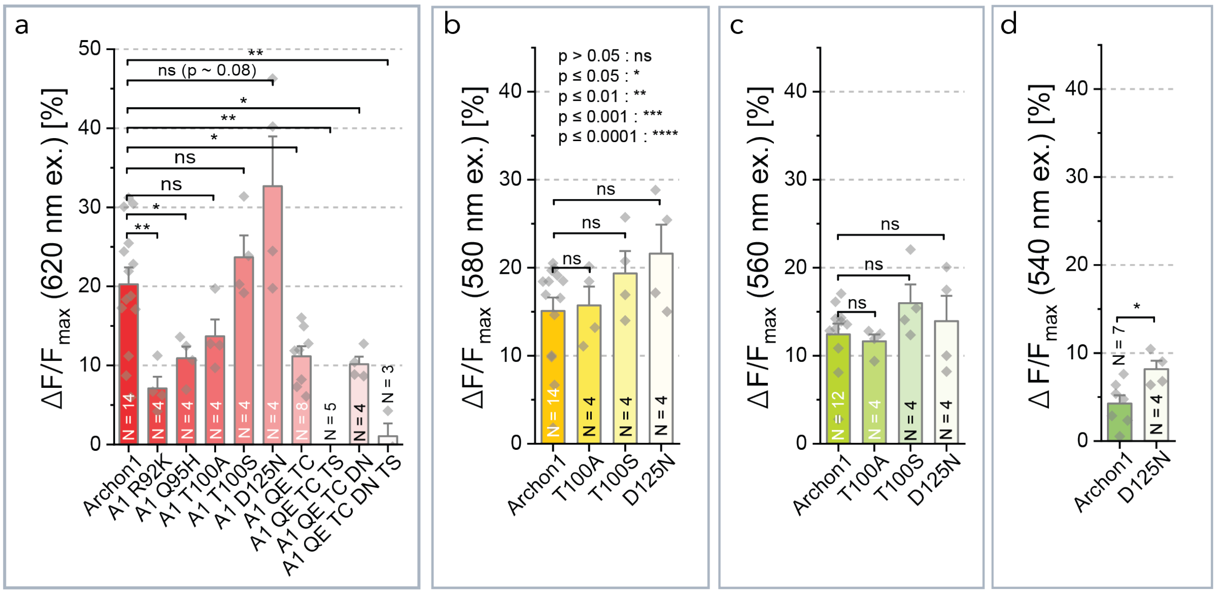

Supplementary Fig. 22 Statistical analysis of the recorded voltage sensitivity of Archon1 variants

Statistical testing on datasets obtained on ND7/23 cells. The maximal ΔF/F observed for each variant at four different excitation wavelengths (620 (a), 580 (b), 560 (d) and 540 (d) nm respectively). Each grey diamond represents the data derived from the individual voltage-clamp recordings. Error is depicted as S.E.M.; p-value determination was done via a Wilcoxon–Mann–Whitney test (two-sided). The p-value definitions are as shown in panel (b). The exact p-values of each construct are summarized in the supplementary table below Table 3. Statistical data plotting and analysis was performed using the “Estimation Statistics” online tool[17]. Source data are provided in the Source Data file (Figure S22).

Supplementary Table 3 Statistical data extracted from the estimation statistics analysis using Wilcoxon–Mann–Whitney U test (two-sided).

620 nm ex.	control_group	test_group	control_N	test_N	effect_size	p-value (Wilcoxon–Mann–Whitney U test (two-sided))
1	Archon1	A1 R92K	14	4	mean difference	0.01687251
2	Archon1	A1 Q95H	14	4	mean difference	0.04945144
3	Archon1	A1 T100A	14	4	mean difference	0.18434810
4	Archon1	A1 T100S	14	4	mean difference	0.36669033
5	Archon1	A1 D125N	14	4	mean difference	0.07972550
6	Archon1	A1 QE TC (ARies1)	14	8	mean difference	0.01048338
7	Archon1	A1 QE TC TS	14	3	mean difference	0.00140278
8	Archon1	A1 QE TC DN	14	4	mean difference	0.03837024

9	Archon1	A1 QE TC DN TS	14	3	mean difference	0.00980143
**580 nm ex.**						
1	Archon1	A1 T100A	14	4	mean difference	0.87343388
2	Archon1	A1 T100S	14	4	mean difference	0.31302784
3	Archon1	A1 D125N	14	4	mean difference	0.22197746
**560 nm ex.**						
1	Archon1	A1 T100A	14	4	mean difference	0.17041812
2	Archon1	A1 T100S	14	4	mean difference	0.32748768
3	Archon1	A1 D125N	14	4	mean difference	0.64770422
**540 nm ex.**						
1	Archon1	A1 D125N	7	4	mean difference	0.02975807

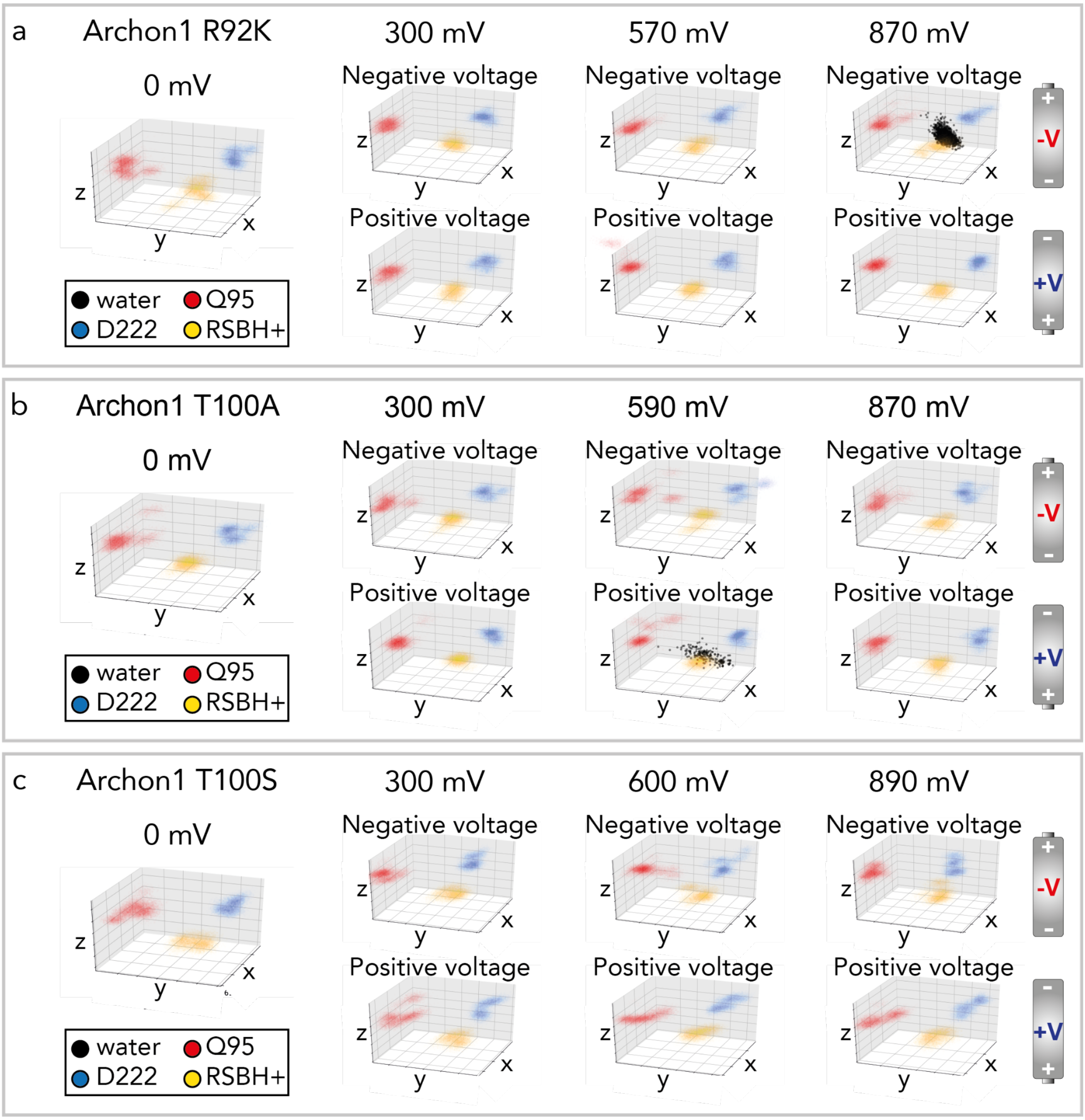

Supplementary Fig. 23 Water occupancy near the RSBH$^+$

Red, blue, and yellow shades indicate Cδ of Q95, Cγ of D222, and the proton at the Schiff base of retinal, respectively. Water oxygens within a sphere of 2 Å centered at the proton of the Schiff base of retinal are represented by black dots. All analysis was conducted after discarding the first half of each 200-ns trajectory to yield equilibrated dynamics. The MD simulations were conducted at 303 K by fixing the protonation states of titratable sites at neutral pH. Source data are provided in the Source Data file (Figure S23).

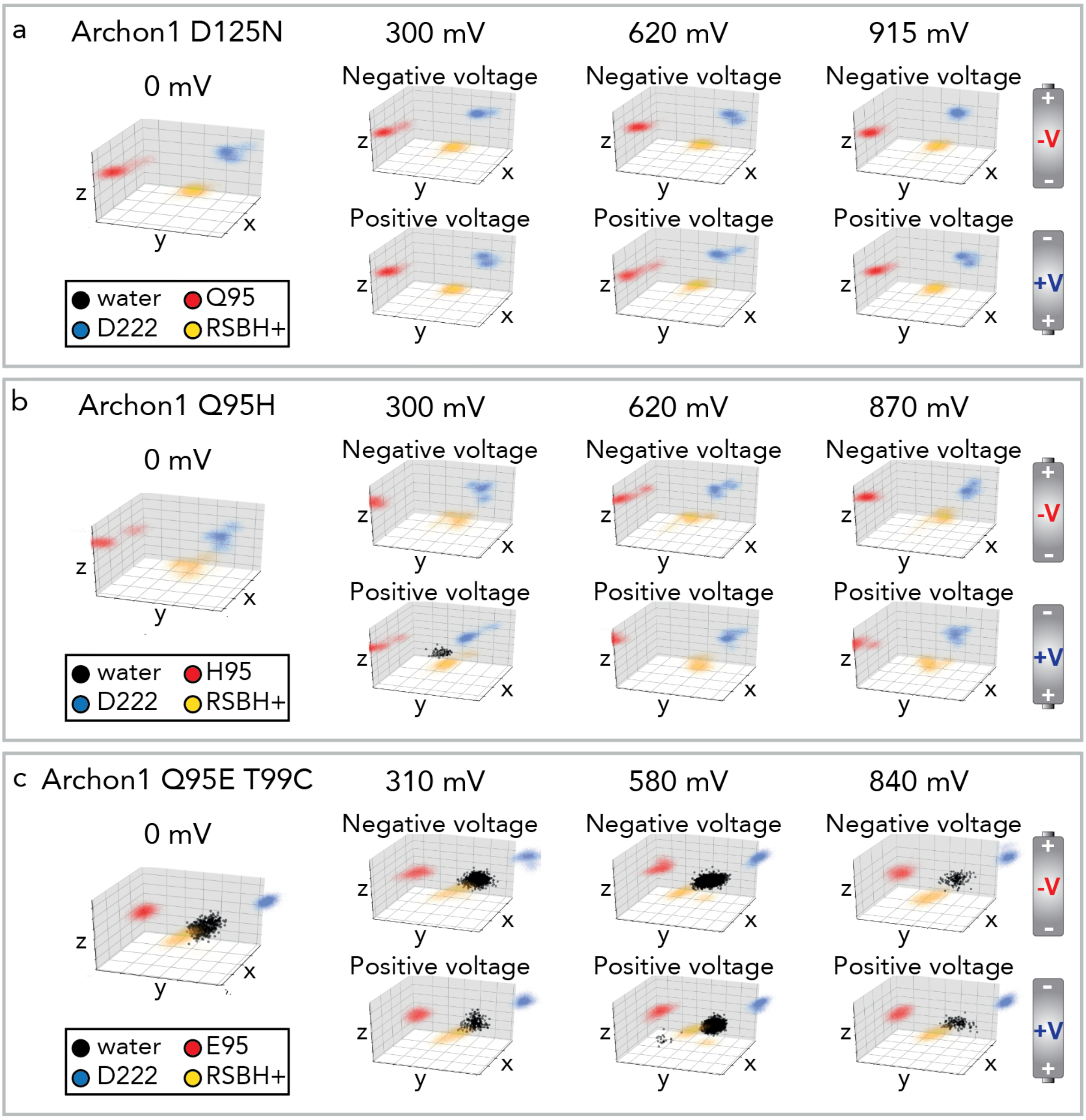

Supplementary Fig. 24 Water occupancy near the RSBH$^+$

Red, blue, and yellow shades indicate Cδ of Q95/E95 (Cγ in the case of H95), Cγ of D222, and the proton at the Schiff base of retinal, respectively. Water oxygens within a sphere of 2 Å centered at the proton of the Schiff base of retinal are represented by black dots. All analysis was conducted after discarding the first half of each 200 ns trajectory to yield equilibrated dynamics. The MD simulations were conducted at 303 K by fixing the protonation states of titratable sites at neutral pH. Source data are provided in the Source Data file (Figure S24).

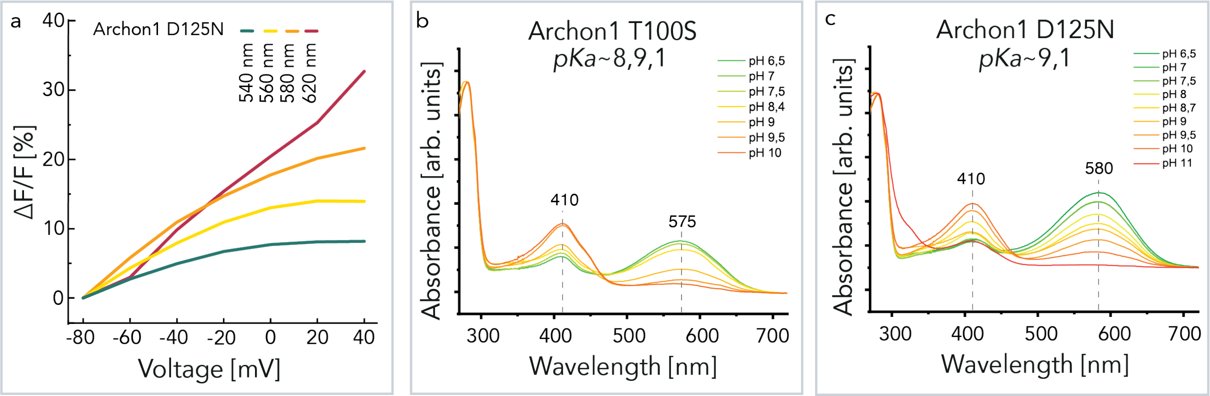

Supplementary Fig. 25 pH titration of variants with increased voltage sensitivity

Panel (a) shows voltage-sensitive fluorescence change (ΔF/F) of Archon1-D125N dependence on an excitation wavelength. The presented excitation wavelengths are 540 nm (green, n=4, p-value 0.030), 560 nm (yellow, n=4, p-value 0.648), 580 nm (orange, n=4, p-value 0.0699) and 620 nm (red, n=4, p-value 0.222, Supplementary Fig. 22, Supplementary Table 3).

Alkaline pH titration of Schiff base pK_a of Archon-T100S (b) and Archon1-D125N (c) variants with improved voltage sensitivity. Source data are provided in the Source Data file (Figure S25b, c).

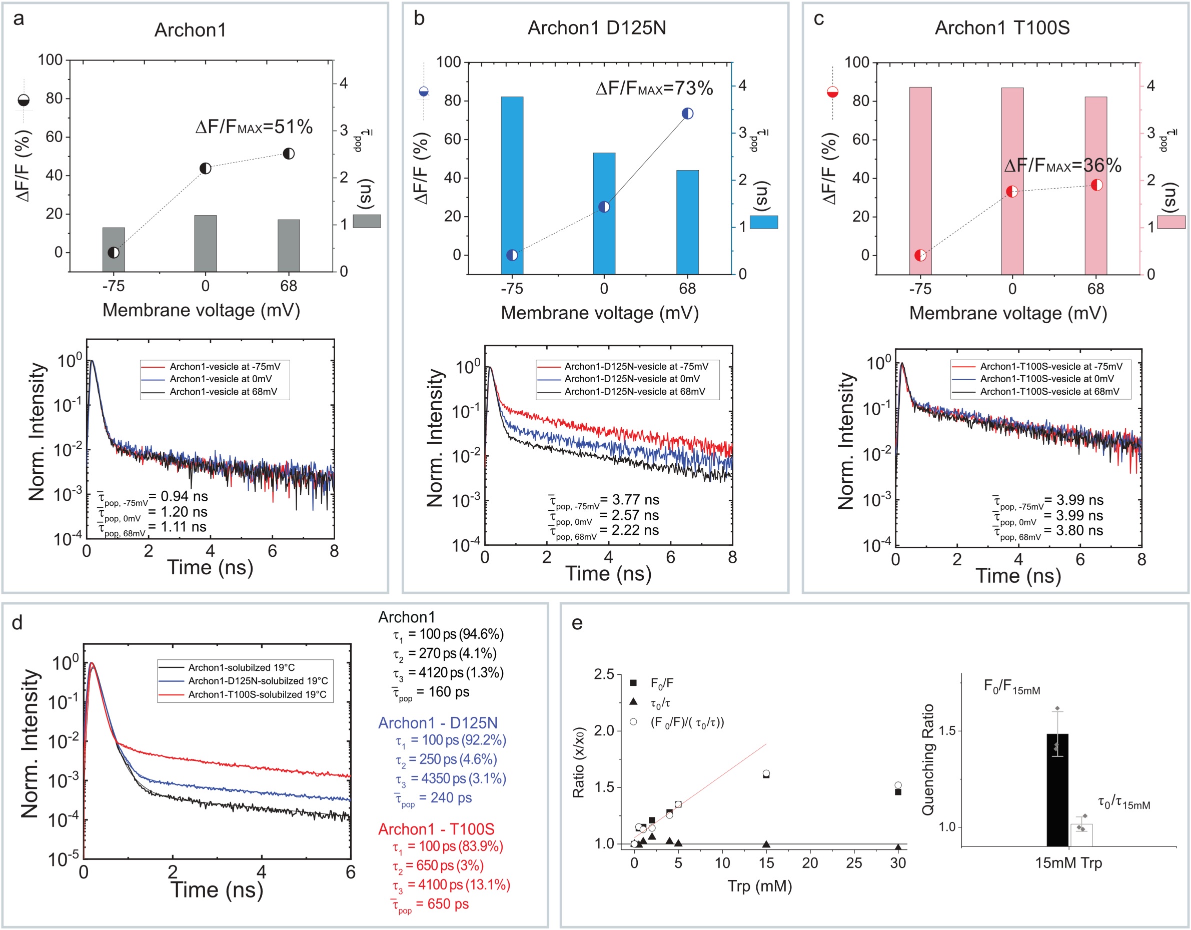

Supplementary Fig. 26 FLIM of Archon1, and the variants D125N, and T100S in solution and in proteoliposomes

(a-c) Voltage sensing in Archon1, D125N, and T100S. Comparison of fluorescence intensity and fluorescence (excited-state) decays at three different membrane voltages as measured in a proteoliposome-FLIM-based assay of purified and vesicle-incorporated Archon1 (a) wild type with fluorescence lifetime fit parameters of 100 ps (78.1%, 74.6%, 76.1%), 1100 ps (6.0%, 3.6%, 3.9%) and 5000 ps (15.9%, 21.8%, 19.9%), (b) D125N with 90 ps (18.1%, 46%, 56.3%), 1050 ps (18.1%, 10.7%, 6.0%) and 5580 ps (63.8%, 43.3%, 37.7%), and (c) T100S with 100 ps (23.9%, 22.8%, 26.7%), 2500 ps (38.5%, 40.2%, 37.8%) and 8000 ps (37.5%, 37.0%, 35.4%). The fractional amplitudes of the respective components at -75 mV, 0 mV and 68 mV are given in brackets. The corresponding normalized fluorescence decay curves and mean fluorescence lifetimes $\bar{\tau}_{pop}$ (lower panel) measured at RT with an excitation wavelength of 640 nm and the voltage-dependent increase in fluorescence as ΔF/F values (upper panel) are shown. (d) Fluorescence (excited-state) decays of the variants D125N and T100S in detergent solution with an excitation wavelength of 640 nm in comparison to Archon1. The fit parameters are given and indicate that the increase in mean fluorescence lifetime ($\bar{\tau}_{pop}$) is mainly due to the increase in the population of the slowest decay time. (e) Static and dynamic Stern-Volmer plots of Retinal-BSA complex with varying concentrations of

Trp. The quenching ratio at 15 mM Trp (n=3 independent experiments) is compared for static and dynamic quenching and indicates mainly static quenching. Data are presented as mean values +/– S.D. Source data are provided in the Source Data file (Figure S25a-e).

.

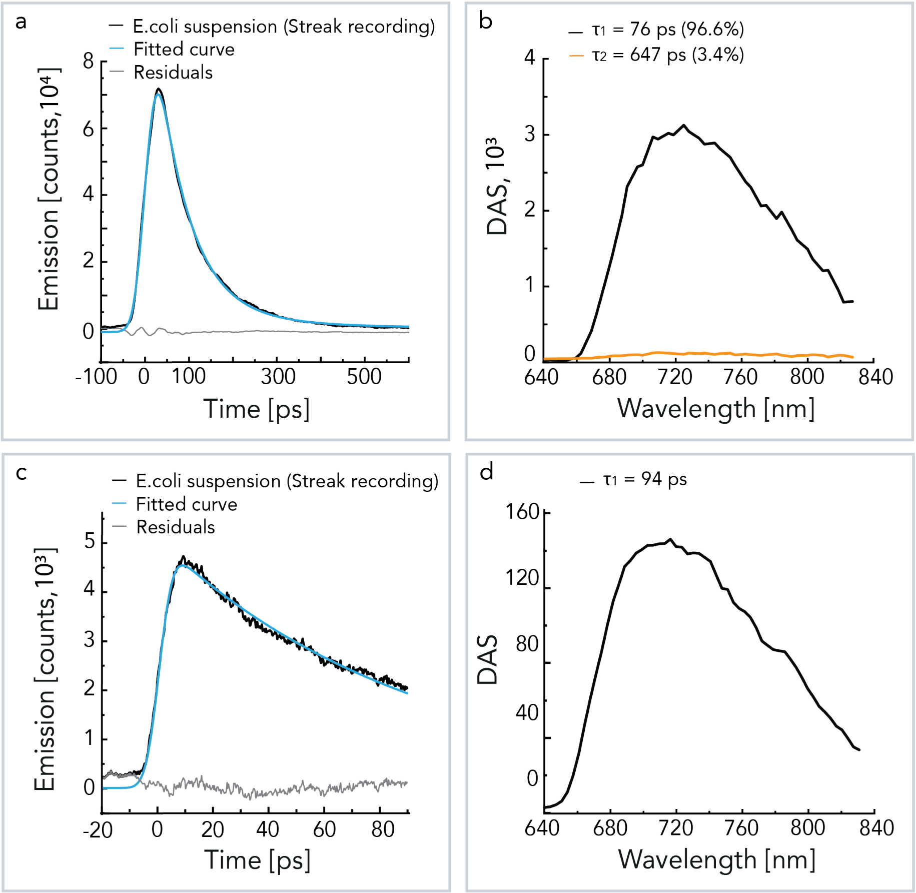

Supplementary Fig. 27 Archon1 fluorescence decay kinetics recorded with streak camera

Fluorescence kinetics of Archon1 measured with a synchroscan streak camera system, which has a time resolution of 14 ps (a,b) or 3-4 ps (c,d), dependent on the applied time base of 1.5 ns (time range 4) or 120 ps (time range 1). As no spectral evolution was observed, the spectral wavelengths were integrated. (a) Kinetics of Archon1 expressed in *E. coli* upon excitation at 625 nm (black line) and fit result (blue line) measured with a time resolution of 14 ps (time base 1.5 ns). The panel (b) shows the resulting DAS of Archon1 in *E. coli* with resolved two time components 76 ps and 647 ps (14 ps resolution). (c) Kinetics of Archon1 expressed in *E. coli* upon excitation at 625 nm (black line), measured with a time resolution of 3-4 ps (time base 120 ps). The panel (d) shows the resulting DAS of time component 94 ps. No fast time component (14 ps), as seen in pump-probe experiments (Supplementary Fig. 7a), is observed here. Source data are provided in the Source Data file (Figure S27).

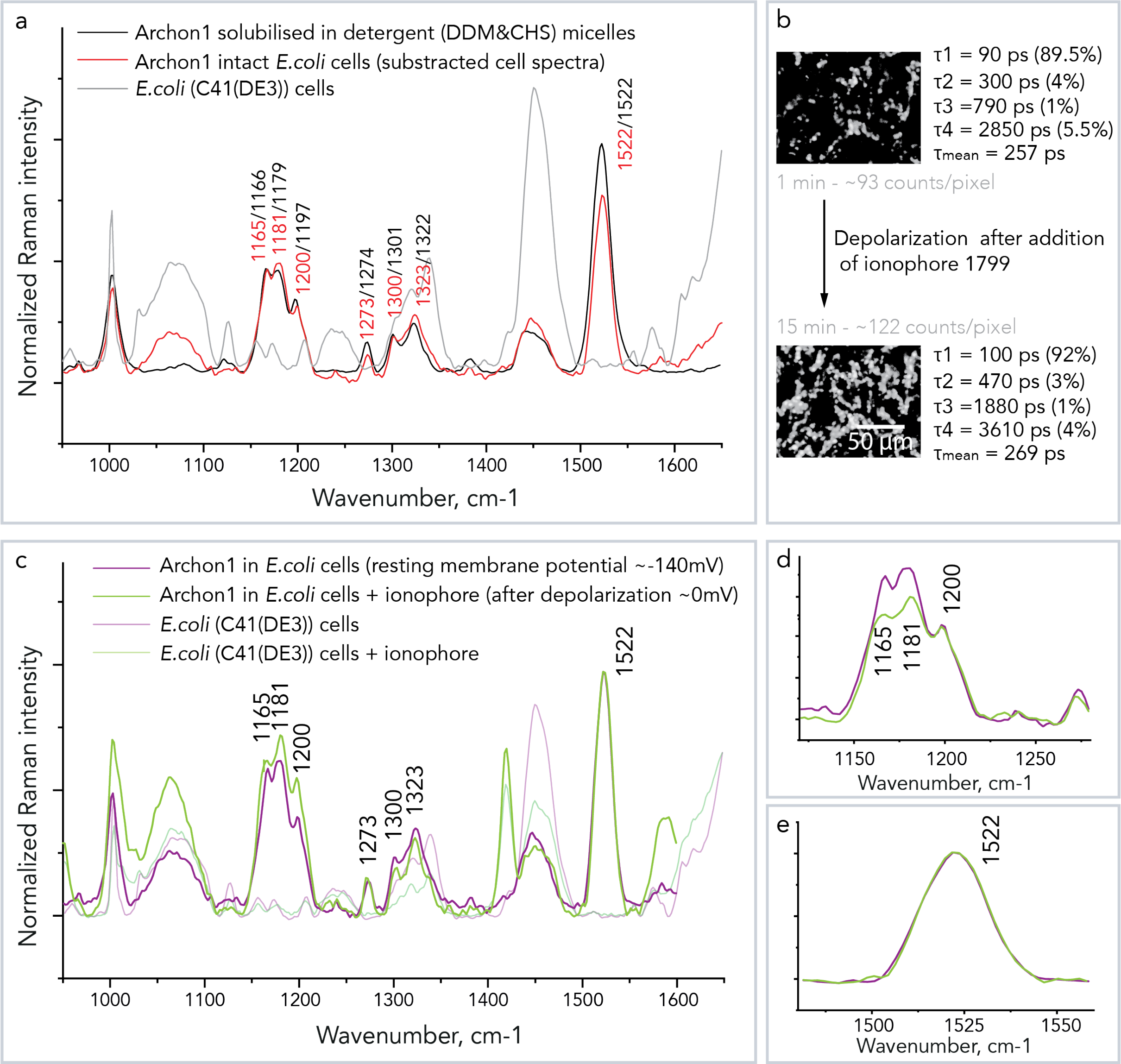

Supplementary Fig. 28 Resonance Raman spectra of Archon1

In panel (a), a comparison of pre-resonance Raman spectra of Archon1 in detergent micelles (black) and E. coli cells (red) is shown. Spectra of *E. coli* cells without expressed Archon1 are shown in grey. Here, we see that the retinal-specific bands in the C-C stretching and C=C stretching regions are preserved and other components of the *E. coli* cells do not contribute significantly to the spectra. (b) Fluorescent lifetime images were measured of *E. coli* cell suspension with overexpressed Archon1 at 1 minute and 15 minutes after addition of the ionophore (1799[18] - 2,6-dihydroxy-1,1,1,7,7,7-hexafluoro-2,6-bis(trifluoromethyl)heptane-4-one). Despite the increase in fluorescence intensity, the mean fluorescence lifetime has not been affected. Fluorescence excitation was at 640 nm; the emission was collected using a long pass filter > 665nm. (c-e) RR spectra of *E. coli* cells with overexpressed Archon1. (c) The Raman spectra recorded in cells give insights into the chromophore structural changes upon membrane depolarization. The purple curve shows the Raman spectrum of Archon1 overexpressed in intact *E. coli* at resting membrane potential (~ −140 mV). The green curve represents

the Raman spectrum of Archon1 overexpressed in *E. coli* 15 min after addition of ionophore (1mM 1799) at abolished membrane potential (0 mV). The spectra are normalized to the peak at 1521 cm^{-1} in the C=C stretching region. Close-up views of the C-C stretching (fingerprint) region (d), and the C=C stretching region (e) are shown in extra panels. There is no change observed in the C=C stretching region, suggesting there is no appreciable change in the absorption spectra, but there are differences in the fingerprint region (d), which might be associated either with equilibrium change between retinal isomers (all-*trans* and 13-*cis*). Upon normalization to the 1200 cm^{-1} peak, which is assigned to all-trans retinal, we note a decrease in intensity of the peaks at 1165 and 1181 cm^{-1}, which are assigned to 13-*cis*-15-*syn* retinal isomer[10,11]. It is likely that upon more positive membrane potential the contribution of all-trans retinal isomer increases, which might result from a voltage-controlled equilibrium of retinal isomers or voltage-controlled equilibrium of protonated/deprotonated 13-*cis*-15-*syn* retinal isomer as seen for Arch3-D95N[12].

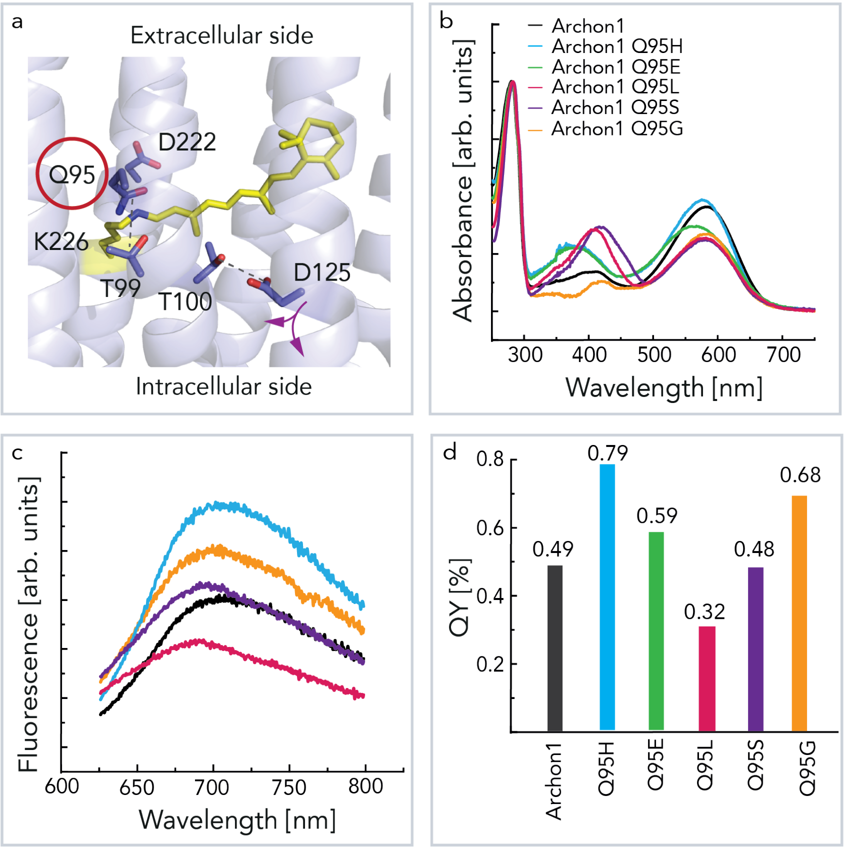

Supplementary Fig. 29 Mutations at the counterion position

(a) Retinal binding pocket with indicated key residues. The absorption spectra (b) and fluorescence emission (c) are shown for Archon1 and its mutants, which carry different counterion D95X mutations. The resulting fluorescence QY is shown in (d). The fluorescence QY is determined upon excitation at wavelength, which corresponds to absorption maxima. Different residues result in shifts

in absorption spectra, pK_a of the Schiff base, and fluorescence QY. Source data are provided in the Source Data file (Figure S29).

Supplementary Table 4 Summary of absorption maximum and estimated fluorescence QY (compared at 580 nm excitation) of the constructs in this study

Construct	λ_{MAX}, nm	QY, %
QuasAr1[5]	590	0.18 (reported- 0.4%)
QuasAr2[5]	580	0.28 (reported- 0.8%)
Archon1[7]	585	0.49
Archon1 – R92K	580	0.74
Archon1 – T100S	575	0.67
Archon1 – D125N	580	0.62
Archon1 – Q95H	575	0.79
Archon1 – Q95E	570	0.59
Archon1 – T99C	604	0.45
Archon1 – Q95E T99C (ARies1)	603	0.61
Archon1 – Q95L	582	0.32
Archon1 – Q95S	583	0.48
Archon1 – Q95G	583	0.68
Archon1 – D222E	574	0.48

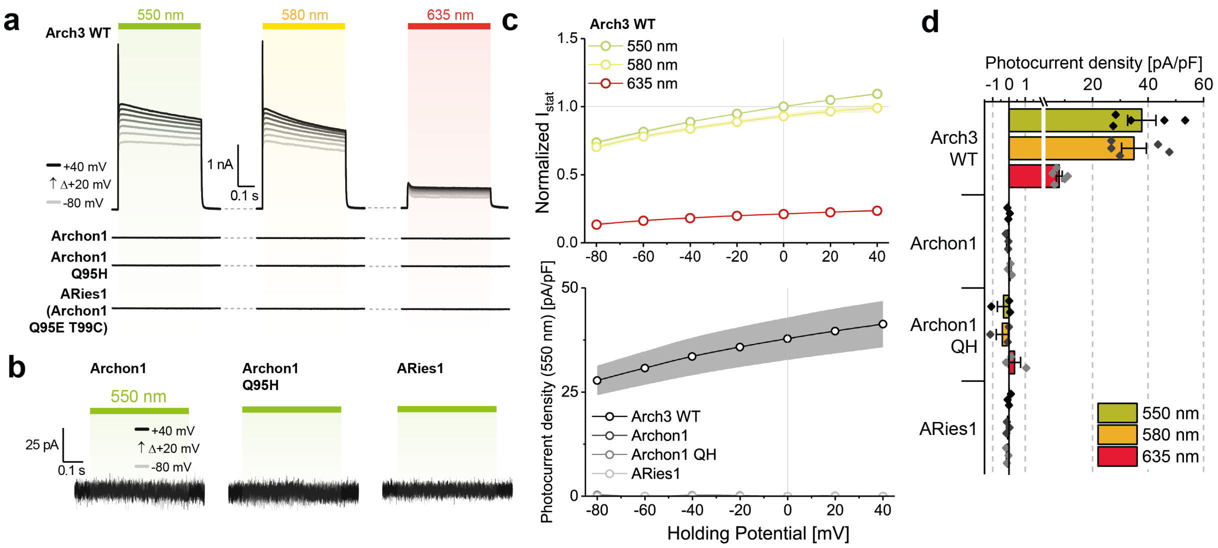

Supplementary Fig. 30 Comparison of photocurrent recordings for Arch3, Archon1 counterion mutants Q95H and Q95E T99C

(a) Comparison of exemplary photocurrents for Arch3 and Archon1 derivatives. (b) Zoom-in on recorded photocurrents of Archon1, Archon1-Q95H and Aries. (c) Upper panel: Normalized stationary photocurrents at the denoted excitation wavelengths for Arch3 WT, normalized to photocurrents at 550 nm excitation and 0 mV holding potential (N=5). Lower panel: Photocurrent densities for Arch3 WT (N=5), Archon1 (N=3), Archon1 Q95H (N=3) and ARies1 (N=3). (d) Photocurrent densities for all variants under all conditions tested. Data are presented as mean values +/- S.E.M. Source data are provided in the Source Data file (Figure S30).

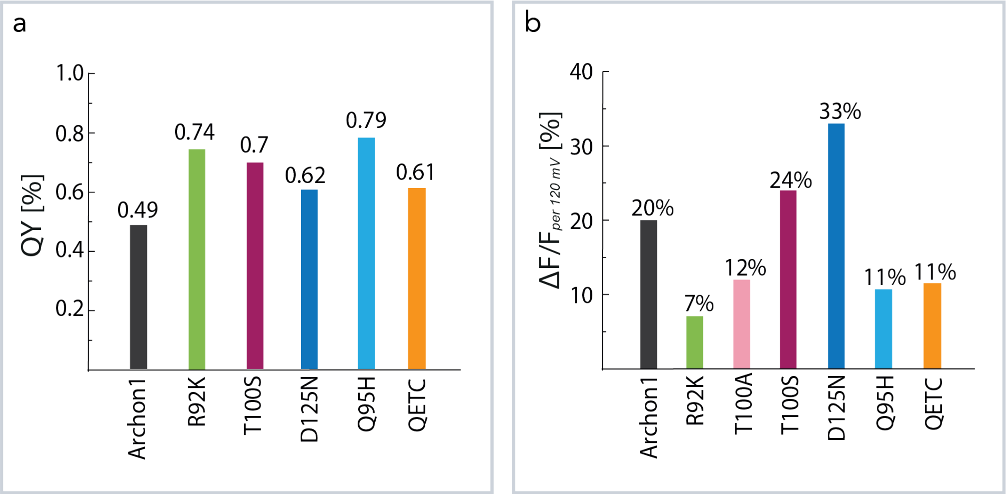

Supplementary Fig. 31 Comparison between Archon1 and its variants

(a) Comparison between the fluorescence QY of Archon1 and its variants. (b) Comparison between the fluorescence intensity change (ΔF/F, %) of Archon1 and its variants upon membrane voltage increase from –80 mV and +40 mV.

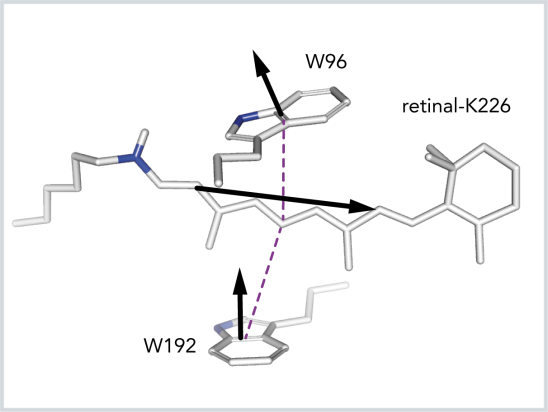

Supplementary Fig. 32 Geometric parameters for calculations of RSBH$^+$-W96 and RSBH$^+$W192 interactions

Inter-vector angle between the vector from C14 to C8 of RSBH$^+$ and the normal of the indole group of W96/W192 was calculated. Distance of RSBH$^+$ - W96/W192 was measured between the center of mass of the polyene chain from C14 to C8 of RSBH$^+$ and the center of mass of the indole group of W96/W192. The vectors and the distances are represented by the black arrows and the purple dotted lines, respectively.

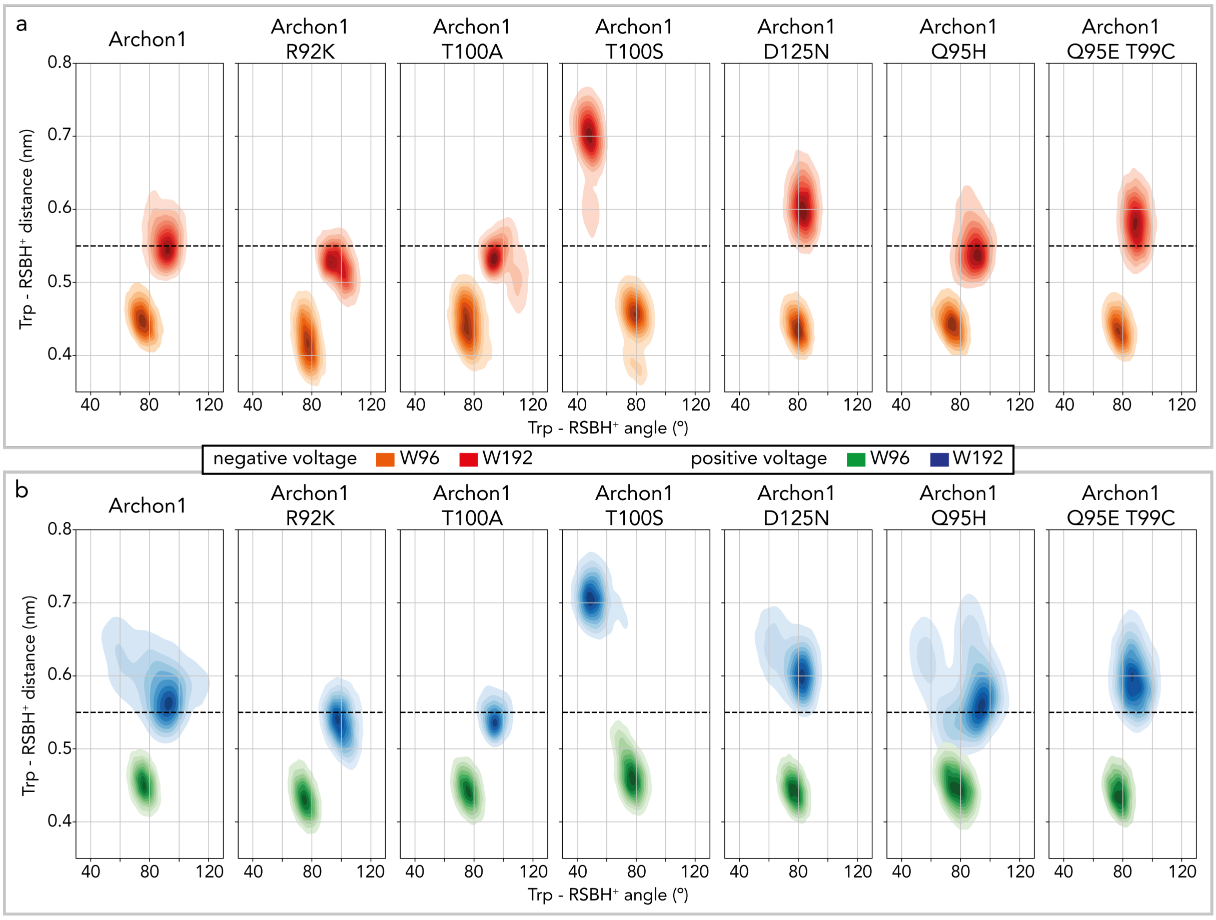

Supplementary Fig. 33 Probability distributions of inter-vector angle and distance between RSBH$^+$ and W96/W192

Inter-vector angle and distance between RSBH$^+$ and W96/W192 as defined in Supplementary Fig. 32 were calculated. (a) Probability distributions of RSBH$^+$-W96 and RSBH$^+$-W192 interactions at negative voltages (charge imbalance of 6 e^-) are depicted as orange and red contour plots, respectively. (b) Probability distributions of RSBH$^+$-W96 and RSBH$^+$-W192 interactions at positive voltages (charge imbalance of 6 e^-) are depicted as green and blue contour plots, respectively. The reasonable distance criterion of tryptophan quenching (5.5 Å) is depicted as a dotted black line. This analysis was conducted after discarding the first half of each 200-ns trajectory to yield equilibrated dynamics. The MD simulations were conducted at 303 K by fixing the protonation states of titratable sites at neutral pH. Source data are provided in the Source Data file (Figure S33).

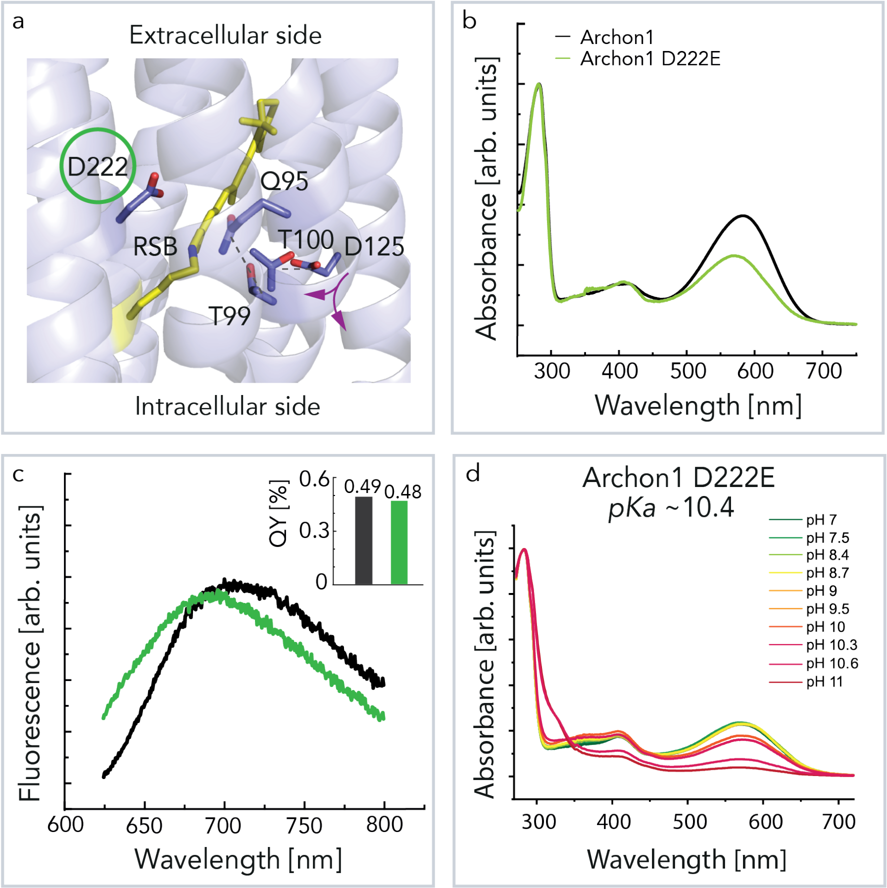

Supplementary Fig. 34 Archon1-D222E study

(a) Retinal binding pocket with indicated key residues. The absorption spectra (b) and fluorescence emission (c) are shown for Archon1 and D222E variant. (d) pH titration of the Schiff base pK_a. Source data are provided in the Source Data file (Figure S34).

Supplementary Table 5 Summary of computational electrophysiology simulations

The MD simulations were conducted at 303 K by fixing the protonation states of titratable sites at neutral pH.

System	Charge imbalances (average membrane voltage ± standard deviation, mV)	Number of K^+, Cl^-, water, lipid	Ion concentration (mM)	Total production simulation time (μs)
Arch3	0 e^-, 2 e^-, 4 e^-, 6 e^- (0, 282±53, 532±28, 787±43)	150, 142, 29856, 500	271	4.8
Archon1	0 e^-, 2 e^-, 4 e^-, 6 e^- (0, 243±49, 551±43, 847±29)	146, 142, 29894, 496	267	4.8
Archon1-R92K	0 e^-, 2 e^-, 4 e^-, 6 e^- (0, 298±25, 570±6, 873±25)	146, 142, 29892, 496	267	4.8
Archon1-T100A	0 e^-, 2 e^-, 4 e^-, 6 e^- (0, 302±17, 592±20, 871±19)	146, 142, 29892, 496	267	4.8
Archon1-T100S	0 e^-, 2 e^-, 4 e^-, 6 e^- (0, 304±19, 600±12, 889±14)	146, 142, 29898, 496	267	4.8
Archon1-D125N	0 e^-, 2 e^-, 4 e^-, 6 e^- (0, 303±22, 620±31, 915±20)	144, 142, 29900, 496	265	4.8
Archon1-Q95H	0 e^-, 2 e^-, 4 e^-, 6 e^- (0, 299±25, 620±20, 873±33)	146, 142, 29894, 496	267	4.8
Archon1-Q95E-T99C (ARies1)	0 e^-, 2 e^-, 4 e^-, 6 e^- (0, 311±18, 583±40, 838±30)	148, 142, 29870, 496	269	4.8

References

1. Kralj, J. M., Douglass, A. D., Hochbaum, D. R., Maclaurin, D. & Cohen, A. E. Optical recording of action potentials in mammalian neurons using a microbial rhodopsin. *Nat. Methods* **9**, 90–95 (2011).

2. Gong, Y., Li, J. Z. & Schnitzer, M. J. Enhanced Archaerhodopsin Fluorescent Protein Voltage Indicators. *PLoS One* **8**, 1–10 (2013).

3. Flytzanis, N. C. *et al.* Archaerhodopsin variants with enhanced voltage-sensitive fluorescence in mammalian and Caenorhabditis elegans neurons. *Nat. Commun.* **5**, (2014).

4. McIsaac, R. S. *et al.* Directed evolution of a far-red fluorescent rhodopsin. *Proc. Natl. Acad. Sci. U. S. A.* **111**, 13034–13039 (2014).

5. Hochbaum, D. R. *et al.* All-optical electrophysiology in mammalian neurons using engineered microbial rhodopsins. *Nat. Methods* **11**, 825–833 (2014).

6. Adam, Y. *et al.* Voltage imaging and optogenetics reveal behaviour-dependent changes in hippocampal dynamics. *Nature* **569**, 413–417 (2019).

7. Piatkevich, K. D. *et al.* A robotic multidimensional directed evolution approach applied to fluorescent voltage reporters article. *Nat. Chem. Biol.* **14**, 352–360 (2018).

8. Kouyama, T. *et al.* Structure of archaerhodopsin-2 at 1.8Å resolution. *Acta Crystallogr. Sect. D Biol. Crystallogr.* **70**, 2692–2701 (2014).

9. Maclaurin, D., Venkatachalam, V., Lee, H. & Cohen, A. E. Mechanism of voltage-sensitive fluorescence in a microbial rhodopsin. *Proc. Natl. Acad. Sci.* **110**, 5939–5944 (2013).

10. Smith, S. O. *et al.* Vibrational Analysis of the all-trans-Retinal Chromophore in Light-Adapted Bacteriorhodopsin. *J. Am. Chem. Soc.* **109**, 3108–3125 (1987).

11. Smith, S. O., Pardoen, J. A., Lugtenburg, J. & Mathies, R. A. Vibrational Analysis of the 13-cis-Retinal Chromophore in Dark-Adapted Bacteriorhodopsin. *J. Phys. Chem.* **91**, 4, 804–819 (1987). doi:10.1021/j100288a011

12. Saint Clair, E. C. *et al.* Near-IR resonance Raman spectroscopy of archaerhodopsin 3: Effects of transmembrane potential. *J. Phys. Chem. B* **116**, 14592–14601 (2012).

13. Jumper, J. *et al.* Highly accurate protein structure prediction with AlphaFold. *Nature* **596**, 583–589 (2021).

14. M. Mirdita, S. Ovchinnikov, M. S. ColabFold - Making protein folding accessible to

all. *bioRxiv* (2021). doi:https://doi.org/10.1101/2021.08.15.456425

15. Baek, M. *et al.* Accurate prediction of protein structures and interactions using a three-track neural network. *Science.* **373**, 871–876 (2021).

16. Bada Juarez, J. F. *et al.* Structures of the archaerhodopsin-3 transporter reveal that disordering of internal water networks underpins receptor sensitization. *Nat. Commun.* **12**, 629 (2021).

17. Ho, J., Tumkaya, T., Aryal, S., Choi, H. & Claridge-Chang, A. Moving beyond P values: data analysis with estimation graphics. *Nat. Methods* **16**, 565–566 (2019).

18. Tittor, J., Schweiger, U., Oesterhelt, D. & Bamberg, E. Inversion of proton translocation in bacteriorhodopsin mutants D85N, D85T, and D85,96N. *Biophys. J.* **67**, 1682–1690 (1994).
