# Supplementary material for: QuasAr Odyssey: the origin of fluorescence and its voltage sensitivity in microbial rhodopsins
Source: Nat Commun. 2022 Sep 20;13:5501. doi: 10.1038/s41467-022-33084-4 (PMC9489792; doi:10.1038/s41467-022-33084-4)
Supplement: Supplementary file 3 — Reporting Summary [file 41467_2022_33084_MOESM3_ESM.pdf]

nature portfolio

Corresponding author(s): Arita Silapetere and Tillmann Utesch

Last updated by author(s): Aug 18, 2022

Reporting Summary

Nature Portfolio wishes to improve the reproducibility of the work that we publish. This form provides structure for consistency and transparency in reporting. For further information on Nature Portfolio policies, see our Editorial Policies and the Editorial Policy Checklist.

Statistics

For all statistical analyses, confirm that the following items are present in the figure legend, table legend, main text, or Methods section.

n/a	Confirmed	
☐	☒	The exact sample size (*n*) for each experimental group/condition, given as a discrete number and unit of measurement
☐	☒	A statement on whether measurements were taken from distinct samples or whether the same sample was measured repeatedly
☐	☒	The statistical test(s) used AND whether they are one- or two-sided<br>*Only common tests should be described solely by name; describe more complex techniques in the Methods section.*
☒	☐	A description of all covariates tested
☒	☐	A description of any assumptions or corrections, such as tests of normality and adjustment for multiple comparisons
☐	☒	A full description of the statistical parameters including central tendency (e.g. means) or other basic estimates (e.g. regression coefficient) AND variation (e.g. standard deviation) or associated estimates of uncertainty (e.g. confidence intervals)
☐	☒	For null hypothesis testing, the test statistic (e.g. *F*, *t*, *r*) with confidence intervals, effect sizes, degrees of freedom and *P* value noted<br>*Give P values as exact values whenever suitable.*
☒	☐	For Bayesian analysis, information on the choice of priors and Markov chain Monte Carlo settings
☒	☐	For hierarchical and complex designs, identification of the appropriate level for tests and full reporting of outcomes
☒	☐	Estimates of effect sizes (e.g. Cohen's *d*, Pearson's *r*), indicating how they were calculated

Our web collection on statistics for biologists contains articles on many of the points above.

Software and code

Policy information about availability of computer code

Data collection	Data was collected using the following softwares: UV-Vis absorption spectroscopy (Varian 721 Inc., Palo Alto, USA and UVProbe v2.34 Shimadzu, Kyoto, Japan); UV-Vis flash photolysis (custom software written in visual C++); fs-pump-probe spectroscopy (custom software written in LabVIEW 2015 (version 15.0, 64-bit)); two-photon excitation spectra (Solis 11.9999, Andor Technology, Belfast, United Kingdom, µ-Manager Edelstein et al. 2014); fluorescence emission (FluorEssence 2.5.2 Horiba Instruments Inc., NJ, USA); HPLC (LC LabSolutions, Shimadzu, Kyoto, Japan); FLIM (photons sorting into 1024 time channels by TCSPC modules (SPC-160, Becker & Hickl, Berlin, Germany), Single Photon Counter (Version 9.80, Becker & Hickl, Berlin, Germany); Raman spectra (OPUS, Bruker, Massachusetts, United States); Fluorescence recordings using streak camera (HPDTA 9.0, Hamamatsu, Hamamatsu City, Japan); Fluorescence recordings with holding patch-clamp in ND7/23 cells (µ-Manager Edelstein et al. 2014, pClamp v10.4 Molecular Devices, San Jose, CA, USA); MD simulations (Gromacs 5.1.2 / 5.1.4; Modeller 9.22; PropKa 3.1; PyMol 2.1; AlphaFold2 (Colab Version); RoseTTAFold (references are included in the text)).
Data analysis	Data was analyzed using the following softwares: stationary UV-Vis absorption spectra, fluorescence emission spectra, Raman spectra, HPLC, (Excel 2016 and Origin 9.0, OriginLab Corporation, Massachusetts, USA); transient UV-Vis spectra (MATLAB R2016b, The MathWorks, Natick, MA and Glotaran 1.5.1, Snellenburg et al. 2012); fluorescence recordings using streak camera (Glotaran 1.5.1, Snellenburg et al. 2012 and Origin 9.0, OriginLab, Northampton, MA); two-photon excitation spectra (custom Python 2.7.16 script , Origin 2017); FLIM (self written Software in C++, Brodwolf et al. 2020 Theranostics and Origin 9.0); fluorescence recordings with holding voltage-clamp in ND7/23 cells (ImageJ, Schneidr et al. 2012, Excel 2016 and Origin 9.0); MD simulations (Gromacs 5.1.2 / 5.1.4; MDAnalysis (references are included in the text); Python 3.7.12; Matplotlib 3.4.3; Numpy 1.20.3; Pandas 1.3.4; Seaborn 0.11.2).

For manuscripts utilizing custom algorithms or software that are central to the research but not yet described in published literature, software must be made available to editors and reviewers. We strongly encourage code deposition in a community repository (e.g. GitHub). See the Nature Portfolio guidelines for submitting code & software for further information.

Data

Policy information about availability of data

All manuscripts must include a data availability statement. This statement should provide the following information, where applicable:

- Accession codes, unique identifiers, or web links for publicly available datasets
- A description of any restrictions on data availability
- For clinical datasets or third party data, please ensure that the statement adheres to our policy

The source data underlying the Figures 1-6 and supplementary Figures is provided in the Source Data file.

Field-specific reporting

Please select the one below that is the best fit for your research. If you are not sure, read the appropriate sections before making your selection.

☒ Life sciences ☐ Behavioural & social sciences ☐ Ecological, evolutionary & environmental sciences

For a reference copy of the document with all sections, see nature.com/documents/nr-reporting-summary-flat.pdf

Life sciences study design

All studies must disclose on these points even when the disclosure is negative.

Sample size	We used Wilcoxon–Mann–Whitney test (two-sided) statistics for Archon1 variants whole cell voltage clamp measurements.<br>For spectroscopic and fluorescence investigation purified sample solution or concentrated cell suspension was used.<br>MD simulations of all individual models were repeated six times. In this way rare events were included but statistically weighted.
Data exclusions	No data was excluded. Prior to measurements, a patch quality with membrane resistance >500 MΩ and access resistance was <10 MΩ was determined prior to data collection.
Replication	Voltage-clamped imaging experiments were performed on 3-14 biological replicates arising from at least 2 different transfection-batches. All data collected was included in the provided plots.<br>Taking into account the invariance in spectroscopic (and fluorescence) recordings of purified protein solution or cell suspension (measured with fixed setup), the measurements were carried out as independent measurements, averaged over multiple scans (where applicable - indicated in the methods section).<br>The MD simulations were repeated multiple times as stated in the main text.
Randomization	For whole patch clamp measurements the cells were selected randomly.<br>For spectroscopic and fluorescence recordings the sample was either purified protein or E.coli cell suspension.
Blinding	Blinding was not necessary for data recording of the presented study.

Reporting for specific materials, systems and methods

We require information from authors about some types of materials, experimental systems and methods used in many studies. Here, indicate whether each material, system or method listed is relevant to your study. If you are not sure if a list item applies to your research, read the appropriate section before selecting a response.

Materials & experimental systems

n/a	Involved in the study	
☒	☐	Antibodies
☐	☒	Eukaryotic cell lines
☒	☐	Palaeontology and archaeology
☒	☐	Animals and other organisms
☒	☐	Human research participants
☒	☐	Clinical data
☒	☐	Dual use research of concern

Methods

n/a	Involved in the study	
☒	☐	ChIP-seq
☒	☐	Flow cytometry
☒	☐	MRI-based neuroimaging

Eukaryotic cell lines

Policy information about cell lines

Cell line source(s)	ND7/23 (SKU: 92090903-1VL; Sigma-Merck)
Authentication	The cell line (ND7/23) used in this study were obtained and authenticated from a vendor. The vendor and the catalog number is listed above. The cell lines used in this study is not listed in the misidentified cell line list of the International Cell Line Authentication Committee. No further authentication was carried out.
Mycoplasma contamination	Tested by vendors, no additional tests were carried out.
Commonly misidentified lines (See ICLAC register)	No commonly misidentified cell lines were used in this study.
